# Gene expression profiling at birth characterizing the preterm infant with early onset infection

**DOI:** 10.1007/s00109-016-1466-4

**Published:** 2016-08-30

**Authors:** Anne Hilgendorff, Anita Windhorst, Manuel Klein, Svetlin Tchatalbachev, Christine Windemuth-Kieselbach, Joachim Kreuder, Matthias Heckmann, Anna Gkatzoflia, Harald Ehrhardt, Josef Mysliwietz, Michael Maier, Benjamin Izar, Andre Billion, Ludwig Gortner, Trinad Chakraborty, Hamid Hossain

**Affiliations:** 1 Department of Neonatology, Grosshadern, Ludwig-Maximilian University Munich, Germany and the Comprehensive Pneumology Center, Helmholtz Zentrum Muenchen, Munich, Germany, Member of the German Center for Lung Research (DZL), Munich, Germany; 2 Department of Pediatrics and Neonatology, Justus-Liebig University Giessen, Germany, Member of the German Center for Lung Research (DZL), Giessen, Germany; 3 Institute for Medical Microbiology, Justus-Liebig University Giessen, Member of the German Center for Infection Research (DZIF), Schubertstr. 81, 35392 Giessen, Germany; 4 Institute for Medical Informatics, Justus-Liebig-University Giessen, Giessen, Germany; 5 Hospital Barmherzige Brueder, Regensburg, Germany; 6 Institute of Medical Biometry and Epidemiology, Philipps-University Marburg, Marburg, Germany; 7 Department of Neonatology and Pediatric Intensive Care, University Medicine, Greifswald, Germany; 8 Institute for Molecular Immunology, Helmholtz Center Munich, Munich, Germany; 9 Broad Institute of MIT and Harvard, Cambridge, MA USA; 10 Department of Pediatrics and Neonatology, University of Saarland, Homburg, Germany

**Keywords:** Neonatal sepsis, Gene expression profiling

## Abstract

**Abstract:**

Early onset infection (EOI) in preterm infants <32 weeks gestational age (GA) is associated with a high mortality rate and the development of severe acute and long-term complications. The pathophysiology of EOI is not fully understood and clinical and laboratory signs of early onset infections in this patient cohort are often not conclusive. Thus, the aim of this study was to identify signatures characterizing preterm infants with EOI by using genome-wide gene expression (GWGE) analyses from umbilical arterial blood of preterm infants. This prospective cohort study was conducted in preterm infants <32 weeks GA. GWGE analyses using CodeLink human microarrays were performed from umbilical arterial blood of preterm infants with and without EOI. GWGE analyses revealed differential expression of 292 genes in preterm infants with EOI as compared to infants without EOI. Infants with EOI could be further differentiated into two subclasses and were distinguished by the magnitude of the expression of genes involved in both neutrophil and T cell activation. A hallmark activity for both subclasses of EOI was a common suppression of genes involved in natural killer (NK) cell function, which was independent from NK cell numbers. Significant results were recapitulated in an independent validation cohort. Gene expression profiling may enable early and more precise diagnosis of EOI in preterm infants.

**Key message:**

- Gene expression (GE) profiling at birth characterizes preterm infants with EOI.
- GE analysis indicates dysregulation of NK cell activity.
- NK cell activity at birth may be a useful marker to improve early diagnosis of EOI.

**Electronic supplementary material:**

The online version of this article (doi:10.1007/s00109-016-1466-4) contains supplementary material, which is available to authorized users.

## Introduction

Preterm birth (PTB) remains a major problem in perinatal medicine and accounts for 75 % of the perinatal mortality and 50 % of the perinatal morbidity [1]. Early onset infections (EOI) in PTB are associated with significant morbidity and a mortality rate of 15–50 %, especially in very immature preterm infants [2, 3]. Although clinical and experimental studies continue to provide valuable insight into processes leading to EOI and its attendant complications [4, 5], biological pathways relevant to the complex pathophysiology of EOI remain poorly understood. This is reflected in the limitations of early diagnostic markers and the absence of targeted treatment approaches in this high-risk patient cohort. Clinical signs are ambiguous and difficult to interpret in the absence of reliable early biochemical markers [6–8]. The gold standard of blood culture-proven sepsis severely underestimates the rate of severe infections in newborns and especially preterm infants, as the diagnostic approach is complicated by maternal antibiotic therapy and hampered by small blood volumes [2, 9]. As outcome and prognosis of EOI mainly depend on early and efficient treatment, sensitive and specific indicators of EOI are crucial at the earliest stage of disease.

In this prospective cohort study, we applied gene expression profiling from umbilical arterial blood of preterm infants <32 weeks of gestational age to provide comprehensive biological information and identify biological pathways relevant for the development of EOI in order to enable the identification of early diagnostic markers.

## Material and methods

### Patients

Newborn infants <32 weeks gestational age (GA) were prospectively included in this study.

Depending on the availability of diagnostic criteria at birth, infants were pro- or retrospectively excluded when one of the following diagnoses was present: premature rupture of membranes ≥3 weeks prior to birth leading to oligo- or anhydramnios, severe congenital malformations, diagnosis of severe metabolic disorders, prepartum treatment of the mother with cytostatic or immunosuppressive medication other than for lung maturation, and postnatal treatment with corticosteroids in a dose ≥1 mg/kg body weight. Analysis of C-reactive protein (CRP), whole white blood count (WBC), and microbiological examination of blood cultures, swabs, urine, and stool samples were carried out in the first 72 h of life. Patients were clinically re-evaluated in short intervals and continuously monitored for vital signs, i.e., heart rate, blood pressure, microcirculation, and breathing pattern.

Patients were allocated to one of the two following groups: (I) EOI and (II) non-EOI (no signs of infection in the first 72 h of life). EOI was diagnosed if the infant showed both a pathologic ratio of immature to total granulocytes (IT ratio ≥ 0.2 as determined by manual counts) and/or a pathologic white blood cell count paralleled or followed by an increase in CRP ≥ 6 mg/l in the first 72 h of life [7, 10–14]. These laboratory signs had to be accompanied by at least three of the following clinical signs suggestive of bacterial infection in the new born infants: pallor, gray skin color, capillary refill >3 s, requiring volume resuscitation or substitution of any catecholamines, dyspnea, tachypnea, requiring respiratory support or supplemental oxygen, increased thermal instability, unexplained hypo- and hyperglycemia, feeding difficulties, bilious reflux and abdominal distension, increasing incidence of apnea and/or bradycardia, lethargy, irritability, and increased or decreased muscle tone [2, 15]. All patients who did not meet the criteria for the EOI were considered as non-EOI. The characteristics and clinical parameters in the first 72 h of life of the patient cohort are given in Table 1 (exploration cohort). *P* values were calculated using Fisher’s exact test for qualitative parameters and Wilcoxon *U* test for quantitative parameters.

**Table 1:** Neonatal characteristics of preterm infants

	EOI	Non-EOI (control)	*P* value
*n*	16	8	
GA (weeks)	29 (24–30)	31 (29–31)	0.008
Birth weight (g)	1085 (590–1730)	1445 (900–1760)	0.046
IUGR	1 (6.25 %)	1 (12.25 %)	1
ANCS	6 (37.5 %)/^a^9 (56.3 %)	6 (75 %)/^a^6 (75 %)	0.667/^a^1
Chorioamnionitis	8 (50 %)	0	0.081
PROM	1 (6.25 %)	0	1
C-section	15 (93.75 %)	8 (100 %)	1
CRIB	6 (1–17)	1 (0–13)	0.102
RDS	16 (100 %)	7 (87.5 %)	0.333
RDS ≥ grade III	9 (56.3 %)	1 (12.5 %)	0.079
IVH	7 (43.75 %)	1 (12.5 %)	0.189
BPD	10 (62.5 %)	0	0.002
Length of mechanical ventilation (days)	7 (3–44)	0 (0–7)	<0.001
ROP	9 (69 %)	4 (50 %)	0.646
Length of hospital stay (days)	70 (10–138)	47 (23–89)	0.065
Death	1 (6.25 %)	0	1
Blood culture positive	1 (6.25 %)	0	1
IT_max_	0.32 (0.11–0.8)^b^	0.17 (0.04–0.37)^b^	0.018
CRP_max_ (mg/dl)	17.8 (7.1–52.3)^b^	4 (4–5.5)^b^	<0.001

Data are given as median and range or percent of total in group. *P* values are calculated using Fisher’s exact test for qualitative parameters and Wilcoxon *U* test for quantitative parameters

*EOI* early onset infection, *GA* gestational age, *IUGR* intrauterine growth restriction, *PROM* premature rupture of membranes, *ANCS* antenatal corticosteroids: complete course including two doses of betamethasone given >24 h prior to birth, last dose <7 days before birth; *CRIB* critical risk index for babies, *RDS* respiratory distress syndrome, *IVH* intraventricular hemorrhage, *BPD* bronchopulmonary dysplasia, *ROP* retinopathy of prematurity

^a^Any ANCS before birth

^b^Value below lower determination threshold (4 mg/dl) was set to be 4 mg/dl

The comprehensive monitoring of the perinatal course is further defined in Supplemental Materials and Methods. The study has been approved by the legal ethical committee (File 79/01, University of Giessen, Germany).

### Blood sampling, RNA isolation, and microarrays

Blood for standard laboratory analyses including WBC and blood samples for transcriptome analyses were obtained from an indwelling umbilical artery catheter immediately after birth. WBCs were repeated upon clinical indication in the later postnatal course as a possible indicator of developing (congenital and nosocomial) infections. Details of the microarray experiments and data analysis can be found in Supplemental Materials and Methods.

Briefly, 250–300 μl of umbilical arterial blood was obtained immediately after birth from an indwelling umbilical artery catheter and directly transferred to 750–900 μl of the PAXgene Blood RNA System (PreAnalytiX, Heidelberg, Germany). RNA isolation was performed according to the manufacturer’s recommendations (PreAnalytiX). RNA was hybridized on CodeLink UniSet Human 10 K Bioarrays (GE Healthcare) using the CodeLink Expression Assay Kit (GE Healthcare) and samples processed using CodeLink Expression Software V4.1 (GE Healthcare).

### Gene expression analysis

In order to account for confounding effects of WBCs on the transcriptome pattern, we evaluated differences between EOI and non-EOI preterm infants in their differential WBCs at birth by using the Wilcoxon rank-sum test. Missing data from WBC counts resulting from technical problems or limited sample size were imputed based on a model using a regularized iterative principal component analysis algorithm [16] taking into account relevant clinical data correlating with WBC, i.e., GA, birth weight, maximum IT ratio, maximum CRP, clinical risk index for babies (CRIB) score, and the presence of respiratory distress syndrome (RDS).

The gene expression dataset was normalized using quantile normalization in R [17]. For statistical analyses of the gene expression data, a rank-based statistics, i.e., Rank Products, was used to identify differentially regulated genes between EOI and non-EOI preterm infants. Being superior to classical and moderated *t* statistics in studies with small sample sizes, this method was chosen for primary analysis [18]. A false discovery rate (FDR) was calculated for each transcript.

To support the results derived from Rank Products and to account for potential hidden confounders affecting gene expression analysis, the data were first corrected for variables that significantly correlated with structural differences between the groups, i.e., the EOI and non-EOI cohort (Table 1). These confounding variables, i.e., gestational age, birth weight, and WBCs, were subsequently taken into account using limma in order to adjust their effect on gene expression analysis. Second, surrogate variable analysis (SVA) [19] was conducted to account for hidden structures in the cohorts, thereby excluding further unknown effects on gene expression analysis (for detailed description see Supplemental Material and Methods). For SVA, two models were compared: the first model corrected gene expression analysis only for the effect of the aforementioned confounders; the second model additionally took the EOI status into account. The identified surrogate variable was used in limma to adjust gene expression analysis. Finally, Rank Products was used to analyze the adjusted data for differential gene expression.

Subsequent statistical analyses were conducted using the software tools “dChip” for hierarchical clustering, “DAVID” for gene ontology, and functional annotation clustering following the software recommendations (Supplemental Materials and Methods).

#### Principal component analysis (PCA)

PCA as a mathematical vector space transformation allows for the reduction of multidimensional data sets to lower dimensions (principle components) accounting for the variability of the data set [20]. PCA was conducted for 292 differentially regulated genes as identified by Rank Products analysis.

#### Disease load index (DLI)

**Fig. 1:**
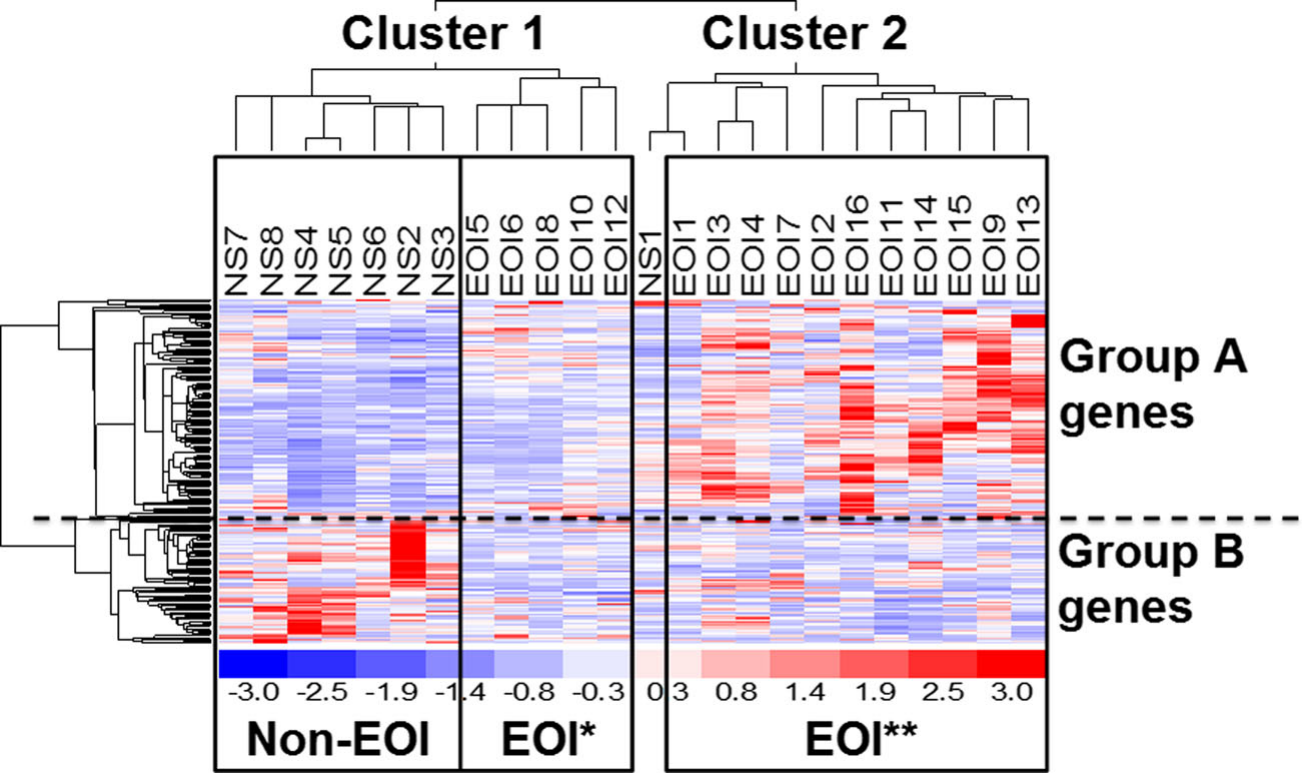
Transcriptional profiles of preterm using hierarchical clustering of differentially expressed genes based on 292 differentially regulated genes. Hierarchical clustering of differentially expressed genes of infants with and without EOI resulted in two main clusters (clusters 1 and 2). Group A genes are upregulated genes in infants with EOI and involved in neutrophil activation, T cell proliferation, hypoxia-induced signaling, and carbohydrate metabolism. Group B genes are downregulated in infants with EOI and mainly involved in NK cell activation. The group of infants with EOI could be further differentiated in a group with low expression of group A genes (EOI*) and EOI with high expression of group A genes (EOI**)

To compare disease-dependent differences in the magnitude of gene expression in preterm infants, we used an aggregate measure designated DLI as described previously [21]. The DLI is a unit-less measure representing the sum of the normalized expression values of defined differentially regulated genes in an individual. Here, the DLI for each infant in clusters 1 and 2 (Fig. 1) was calculated. Subsequently, mean DLIs of each patient group (non-EOI; EOI) were compared and the significant differences between the DLIs of each patient group were given as a *P* value derived from analysis of variances (ANOVA), pairwise Student’s *t* test with Benjamini-Hochberg correction for group A genes as well as the non-parametric Kruskal-Wallis test and the non-parametric pairwise Wilcoxon ranks sum test with Benjamini-Hochberg correction for group B genes. The complete data set is available at the Gene Expression Omnibus (GEO) database under the accession number GSE5760.

### Measurement of NK cell number and activation

Umbilical arterial blood specimens for measurement of natural killer (NK) cell number were collected from a separate cohort of preterm infants (*n* = 20) < 32 weeks of GA included and characterized exactly as described above. Hematopoietic cell staining was performed with a PeCy5.5-labeled mouse IgG1 anti-human CD45 antibody from Invitrogen (Carlsbad, CA, USA) and separation of leukocyte fractions by simultaneously using the following antibodies: PB-labeled mouse IgG1 anti-human CD3, Alx700-labeled mouse IgG1 anti-human CD19 and APC-labeled mouse IgG1 anti-human NKp46 from BD Biosciences (San Jose, CA, USA), APC-Alx750-labeled mouse IgG2a anti-human CD14 from Invitrogen, FITC-labeled mouse IgG 1 anti-human CD15 from Miltenyi Biotec (Bergisch Gladbach, Germany), and corresponding isotype controls. Flow cytometry was performed on a LSRII Flow Cytometer (BD) using the proper controls to set gates and analyzed with FlowJo8.7.1 software. Dead cells were excluded using propidium iodide labeling and doublets by gating on single cells (FSC-H to FSC-W channel).

### Validation and replication of the microarray results by TaqMan RT-PCR

Microarray results were confirmed within the exploration cohort and validated in an independent validation cohort by RT-PCR using TaqMan® technology (Applied Biosystems, Darmstadt, Germany) (Supplemental Materials and Methods).

Briefly, TaqMan quantitative real-time (RT)-PCR was performed for 10 human genes deriving from the microarray results (ANXA1, CD163, GNLY, HIF1A, KLRC2, KLRD1, MPO, PGLYRP1, TNFRSF10A, CD177) and three housekeeping genes glucose-6-phosphate dehydrogenase (G6PD), succinate dehydrogenase complex, subunit A, flavoprotein (Fp) (SDHA), and phosphoglycerate kinase 1 (PGK1) as internal controls for normalization. To test whether the microarray results could be replicated, the gene expression of the same 10 genes was investigated in a validation cohort consisting of 43 new preterm infants by RT-PCR as described in Supplemental Materials and Methods. The patient characteristics of the validation cohort (15 patients with EOI, 28 non-EOI) are given in Supplemental Table 2.

## Results

Thirty very preterm infants were prospectively enrolled in this study. Twenty-four of 30 samples met the high RNA quality criteria and were further processed for microarray analyses; of these, 16 were retrospectively allocated to the EOI cohort based on the presence of clinical parameters for EOI in the first 72 h of life. Eight infants without EOI were assigned to the control group (non-EOI). The characteristics of the patient cohorts are shown in Table 1.

### Gene expression analysis of umbilical arterial blood reveals differential gene expression profiles in preterm infants with EOI at birth

Using a rank-based statistics, comparison of gene expression of infants with and without EOI revealed 292 differentially regulated genes (FDR ≤ 0.1). Of these, 219 genes had significantly higher gene expression levels (upregulated genes) in infants with EOI, while 73 genes had significantly lower expression levels (downregulated genes) (Supplemental Table 1a, b).

The differentially regulated transcripts were involved in processes related to *inflammatory response* (enrichment score (ES) = 5.5), *chemotaxis* (ES 1.9), and *leucocyte activation* (ES 1.8) as well as in catabolic processes (*protein catabolic processes* ES = 1.8, *glycolysis* ES = 1.2) (Supplemental Table 4).

To account for unknown confounders and hidden structures affecting gene expression analysis, the data set was corrected for the variables GA, birth weight, and WBC using SVA and limma. The analysis revealed a considerable overlap of functional categories and the corresponding genes when compared with the results derived from Rank Products only (Supplemental Tables 4 and 5), i.e., the findings obtained from adjusted gene expression data supported the results from the initial Rank Products analysis. Notably, comparison of the differential WBCs at birth showed no significant differences between EOI and non-EOI preterm infants (Table 2).

**Table 2:** White blood count of EOI and non-EOI

White blood cells [×^3^/μl]	EOI			Non-EOI			*P* value
	*N*	Mean	SE	*N*	Mean	SE	
LEU	12	6.92	0.26	8	5.90	0.3	0.589
segNEU	11	3.44	0.38	6	1.29	0.15	0.145
bandNEU	11	2.26	0.28	5	0.30	0.05	0.141
juvNEU	10	0.38	0.03	7	0.37	0.03	0.695
LYM	11	3.48	0.16	6	4.42	0.29	0.248
MON	11	0.96	0.12	6	0.57	0.05	0.960

*P* value from Wilcoxon *U* test

*LEU* leucocytes, *segNEU* segmented neutrophils, *bandNEU* band neutrophils, *juvNEU* juvenile neutrophils, *LYM* lymphocytes, *MON* monocytes, *SE* standard error

### Gene expression profiling reveals two groups of preterm infants with EOI

**Fig. 2:**
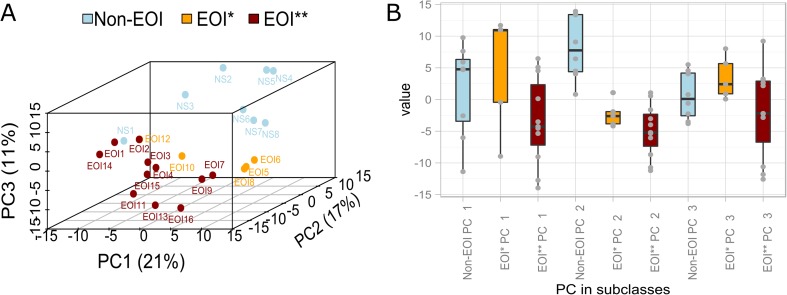
**a** Three-dimensional principal component analysis (PCA) and **b** boxplots of principal components (PC) based on 292 differentially regulated genes. Individual patients are plotted based on their respective positions along the three axes derived from PCA. Patient subclassifications are indicated by *color*. PCA indicates a high degree of separation between the two subclasses EOI* and EOI** and non-EOI

Hierarchical clustering of the differentially regulated genes (FDR ≤ 0.1) identified by Rank Products analysis separated the cohort of preterm infants into clusters 1 and 2 (Fig. 1). Cluster 1 included preterm infants from both groups, EOI (EOI*), as well as non-EOI, while cluster 2 included infants with EOI (EOI**) with one exception. Thus, two subclasses of EOI were identified, designated as EOI*, occurring mainly in cluster 1 and EOI** in cluster 2. The two subclasses of EOI were also identified by PCA which provided a high degree of separation between the two subclasses EOI* and EOI** (Fig. 2a, b).

**Fig. 3:**
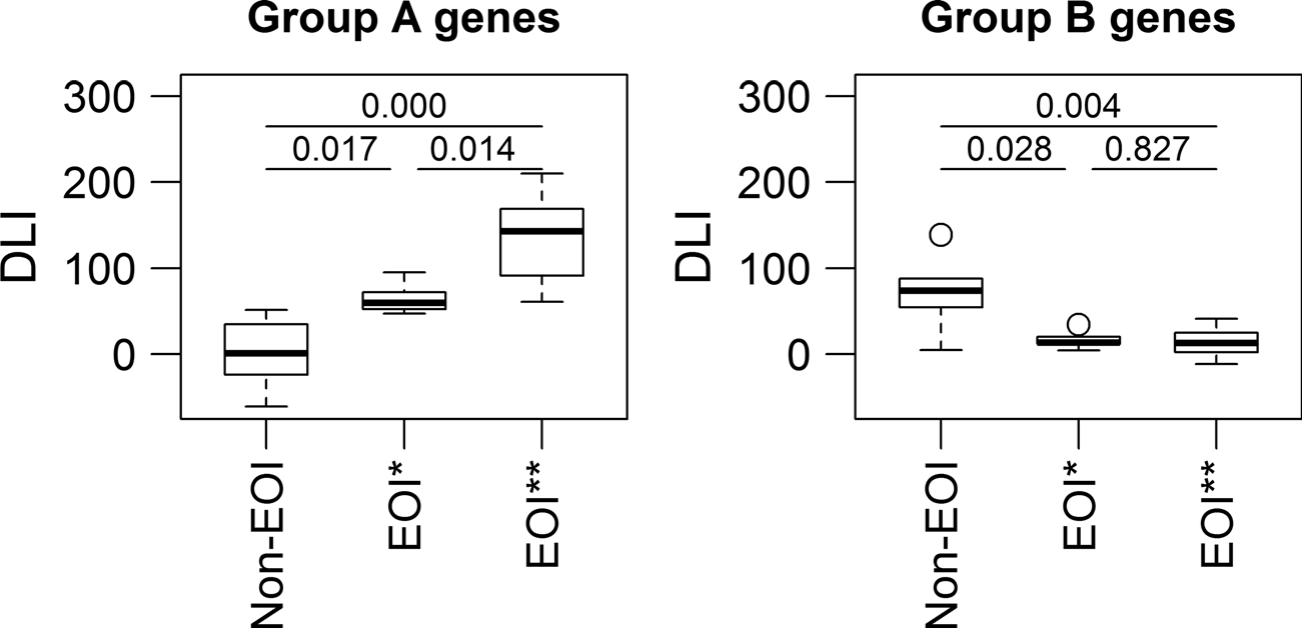
Mean DLIs of differentially regulated group A and B genes in preterm infants with and without EOI. Comparison of disease load indices (*DLIs*) of non-EOI, EOI with low expression of group A genes (EOI*) and EOI with high expression of group A genes (EOI**). Group A genes show significantly higher DLI of group A genes in EOI** compared to EOI* and non-EOI. Group B genes show significantly lower DLI in both EOI* and EOI** compared to non-EOI. The significance of the difference between the DLIs of the patients groups was given as a *P* value deriving from pairwise Student’s *t* test with Benjamini-Hochberg correction for group A genes and from non-parametric Kruskal-Wallis test and non-parametric pairwise Wilcoxon ranks sum test with Benjamini-Hochberg correction for group B genes

The two subclasses EOI* and EOI** were distinguished by the expression of two groups of genes, namely group A and B genes (Fig. 1): Group A genes were overexpressed in EOI** as compared to EOI* and non-EOI and were involved in *neutrophil activation*, *T cell proliferation*, *hypoxia-induced signaling*, and *carbohydrate metabolism*. Group B genes were downregulated in both EOI* and EOI** as compared to non-EOI and were involved in *NK cell activation*. To compare for differences in the magnitude of the gene expression of group A and B genes, the aggregative DLIs of group A and B genes in EOI* and EOI** were determined (Fig. 3): EOI** had a significantly higher DLI for group A genes than both EOI* (*P* = 0.0143) and non-EOI (*P* = 1e-05). Hence, the subclass EOI** activated genes involved in neutrophil activation, T cell proliferation, hypoxia-induced signaling, and carbohydrate metabolism on a higher level than subclass EOI*. For group B genes, no significant differences occurred in the expression level between the subclasses EOI* and EOI** (Fig. 3, *P* = 0.8292). But both EOI* and EOI** showed a significantly lower DLI for group B genes compared to non-EOI (*P* = 0.0280 and *P* = 0.0036, respectively) indicating decreased expression of genes involved in NK cell activation. Clinical variables characterizing EOI* and EOI** subclasses are given in Table 4, showing no statistically significant differences between the groups. However, although not significant, preterm infants in the EOI* group showed more complications than EOI**, i.e., higher RDS > grade III, intraventricular hemorrhage (IVH), and development of bronchopulmonary dysplasia (BPD).

### Increased activation of neutrophils in preterm infants with EOI

**Fig. 4:**
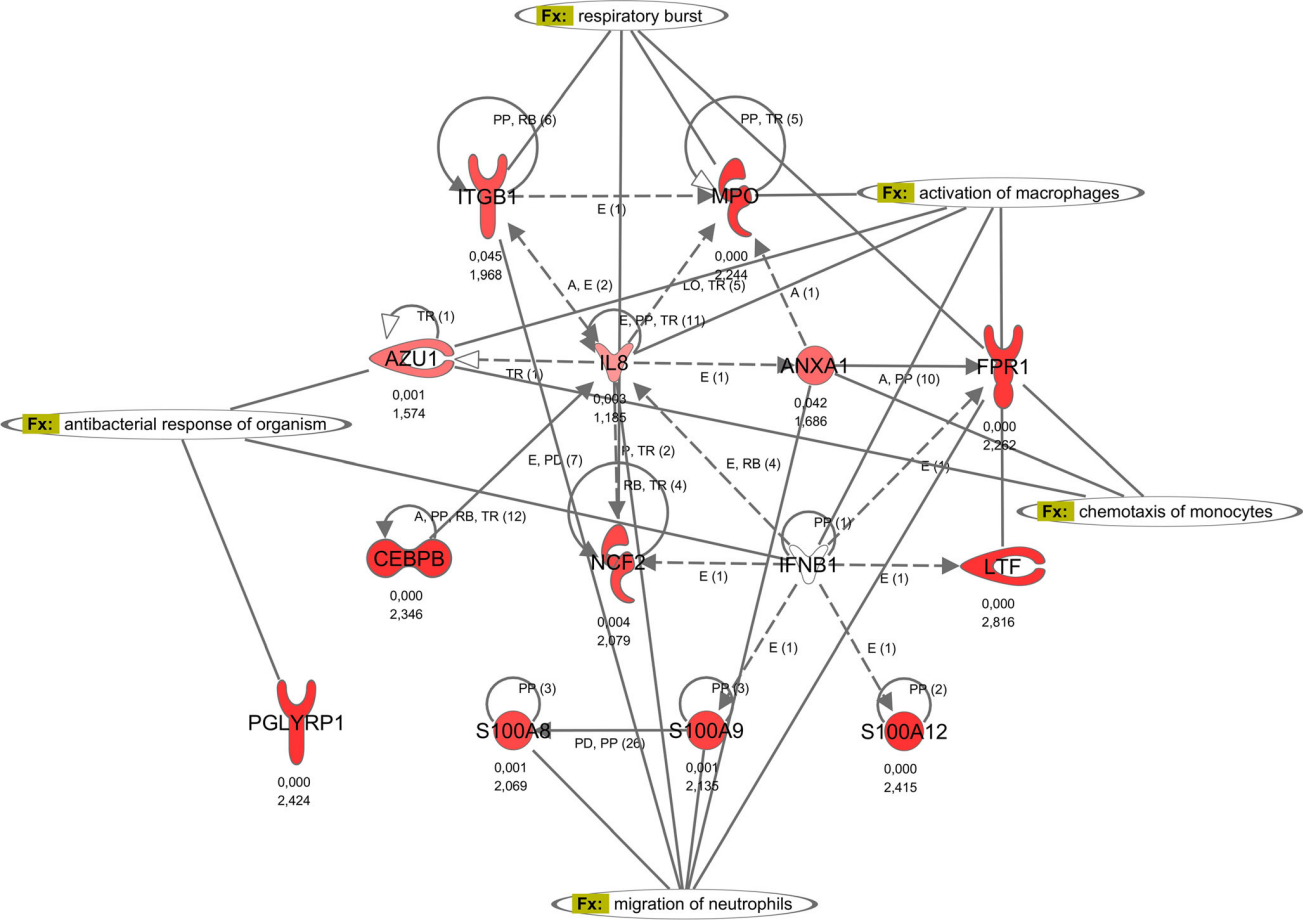
Regulation of neutrophil activation. The gene interaction network *regulation of neutrophil activation* of the differentially regulated genes in EOI shows the interaction between calgranulins (S100A8, S100A9, S100A12) and genes involved in phagocytotic activity, granula secretion, and respiratory burst of neutrophils (e.g., MPO, AZU1, LTF, NCF2). Upregulated genes are depicted in *red* and downregulated genes in *green*. *P* value and fold change are given beneath each gene symbol. Genes with an unknown regulation are depicted in *white*. Relationships and interactions between molecules are abbreviated as follows: *A* activation, *B* binding, *C* causes/leads to, *CC* chemical–chemical interactions, *CP* chemical–protein interactions, *E* expression (includes metabolism/synthesis for chemicals), *EC* enzyme catalysis, *I* inhibition, *L* proteolysis (includes degradation for chemicals), *LO* localization, *M* biochemical modification, *MB* group/complex membership, *P* phosphorylation/dephosphorylation, *PD* protein–DNA interactions, *PP* protein–protein interactions, *PR* protein–RNA interactions, *RB* regulation of binding, *RE* reaction, *RR* RNA-RNA interactions, *T* transcription, *TR* translocation

Neutrophils play a pivotal role in the innate immune response, as they migrate to the site of infection and help limit microbial infections. Increased activity of neutrophils in infants with EOI, especially in subclass EOI**, is indicated by overexpression of group A genes involved in phagocytotic activity, granula secretion, and respiratory burst of neutrophils as depicted in the interaction network in Fig. 4. The increased activation is given by overexpression of phospholipid scramblase 1 (PLSCR1), an enzyme involved in hematopoietic proliferation and differentiation of neutrophils (Table 3). Overexpression of the proinflammatory calgranulins A (S100A8), B (S100A9), and C (S100A12) suggests enhanced neutrophil chemotaxis, adhesion, and migration in infants with EOI. Furthermore, increased expression of CD177, a receptor on the surface of neutrophils, indicated enhanced transmigration. Strong activation of the neutrophils was also reflected by the overexpression of myeloperoxidase (MPO), neutrophil cytosolic factor 2 (NCF2), lactoferrin (LTF), azurocidin 1 (AZU1), peptidoglycan recognition protein 1 (PGLYRP1) as well as cathepsin D (CTSD).

**Table 3:** Selected genes in relevant biological processes

Biological process/gene name	Symbol	Fold change	FDR
Neutrophil function			
Azurocidin 1 (cationic antimicrobial protein 37)	AZU1^a^	1.57	0.001
Catalase	CAT^a^	3.51	0.000
Cathepsin D	CTSD	2.00	0.007
CD177	CD177^a^	3.49	0.000
Formyl peptide receptor 1	FPR1	2.26	0.000
Grancalcin, EF-hand calcium binding protein	GCA^a^	2.79	0.000
Lactotransferrin	LTF^a^	2.82	0.000
Leukotriene A4 hydrolase	LTA4H	2.34	0.003
Myeloperoxidase	MPO	2.24	0.000
Neutrophil cytosolic factor 2 (65 kDa, chronic granulomatous disease, autosomal 2)	NCF2	2.08	0.004
Peptidoglycan recognition protein 1	PGLYRP1^a^	2.42	0.000
Phospholipid scramblase 1	PLSCR1^a^	2.55	0.000
S100 calcium binding protein A8 (calgranulin A)	S100 A8	2.07	0.001
S100 calcium binding protein A9 (calgranulin B)	S100 A9	2.14	0.001
S100 calcium binding protein A12 (calgranulin C)	S100 A12	2.42	0.000
S100 calcium binding protein P	S100P	2.55	0.000
Natural killer cell function			
Killer cell lectin-like receptor subfamily B, member 1	KLRB1	−1.75	0.069
Killer cell lectin-like receptor subfamily C, member 2	KLRC2^a^	−1.64	0.030
Killer cell lectin-like receptor subfamily D, member 1	KLRD1	−1.83	0.053
Granulysin	GNLY^a^	−2.48	0.001
C-type lectin domain family 1, member B	CLEC1B	−1.70	0.036
Solute carrier family 30 (zinc transporter), member 1	SLC30A1	−1.89	0.071
Zinc finger, CCHC domain containing 2	ZCCHC2^a^	−2.14	0.010
Zinc finger E-box binding homeobox 1	ZEB1	−1.72	0.007
Zinc finger protein 839	ZNF839	−1.72	0.089
Zinc finger protein 671	ZNF671^a^	−2.69	0.000
T cell function			
Carcinoembryonic antigen-related cell adhesion molecule 1 (biliary glycoprotein)	CEACAM1	1.78	0.032
Chemokine (C-X-C motif) receptor 4	CXCR4^a^	1.69	0.022
Hematopoietic cell-specific Lyn substrate 1	HCLS1^a^	2.21	0.002
Integrin, beta 1 (fibronectin receptor, beta polypeptide, antigen CD29 includes MDF2, MSK12)	ITGB1	1.97	0.045
Interferon gamma receptor 2 (interferon gamma transducer 1)	IFNGR2^a^	1.88	0.004
Interleukin 10	IL10	1.71	0.022
Protein tyrosine phosphatase, non-receptor type 22 (lymphoid)	PTPN22	2.33	0.003
Protein tyrosine phosphatase, receptor type, C	PTPRC^a^	2.30	0.001
Transcription			
Hypoxia-inducible factor 1, alpha subunit (basic helix-loop-helix transcription factor)	HIF1A	1.75	0.055
CCAAT/enhancer binding protein (C/EBP), beta	CEBPB^a^	2.35	0.000
CCAAT/enhancer binding protein (C/EBP), alpha	CEBPA	2.10	0.002
B-cell CLL/lymphoma 6	BCL6	2.00	0.017
CREB binding protein	CREBBP	−1.95	0.023
GATA binding protein 3	GATA3^a^	−1.84	0.043

^a^Significant in Rank Products and SVA

### Decreased activation of NK cells in preterm infants with EOI

NK cells constitute a component of the innate immune system in combating intracellular pathogens and activating and modulating the adaptive immune response. Activation of NK cells is regulated by the expression of a variety of receptors.

In EOI, the NK cell activating killer cell lectin-like receptors (KLRs) such as KLR subfamily B member 1 (KLRB1), KLR subfamily C member 2 (KLRC2), and KLR subfamily D member 1 (KLRD1) were downregulated (Tables 3 and 4). GNLY, whose expression is regulated via signaling through KLRB1, KLRC2, and KLRD1, is an antimicrobial, cytolytic protein in the granules of NK cells and was also downregulated in EOI.

**Table 4:** Characteristics of preterm infants EOI* and EOI**

	EOI*	EOI**	*P* value
*n*	5	11	
GA (weeks)	28 (24–30)	29 (24–30)	0.910
Birth weight (g)	1060 (700–1390)	1100 (590–1730)	0.610
IUGR	0	1 (9 %)	1
ANCS	3 (60 %)/^a^4 (80 %)	3 (27 %)/^a^5 (55 %)	0.580/^a^0.228
Chorioamnionitis	2 (40 %)	6 (55 %)	1
PROM	0	1	1
C-section	5 (100 %)	10 (91 %)	1
CRIB	2 (1–11)	7 (1–17)	0.351
RDS	5 (100 %)	11 (100 %)	1
RDS ≥ grade III	4 (80 %)	6 (55 %)	0.588
IVH	3 (60 %)	4 (36 %)	0.596
BPD	4 (80 %)	6 (55 %)	0.600
Length of mechanical ventilation (days)	7 (5–44)	7 (3–23)	0.597
ROP	2 (50 %)	7 (78 %)	0.596
Length of hospital stay (days)	74 (51–138)	70 (10–124)	0.844
Blood culture positive	1 (20 %)	0	0.267
IT_max_	0.2 (0.1–0.7)^b^	0.4 (0.1–0.8)^b^	0.733
CRP_max_ (mg/dl)	12.4 (7.7–24.3)^b^	18.9 (7.1–52.3)^b^	0.257
White blood cell counts, mean (standard error)			
LEU	8.32 (0.97)	6.21 (0.32)	0.552
segNEU	3.37 (0.9)	3.46 (0.59)	0.759
bandNEU	4.02 (1.73)	1.6 (0.26)	0.475
juvNEU	0.43 (0.07)	0.35 (0.06)	0.594
LYM	3.32 (0.49)	3.54 (0.24)	1
MON	2.15 (0.77)	0.52 (0.05)	0.126

Data are given as median and range or percent of total in group. *P* values are calculated using Wilcoxon *U* test for quantitative parameters and Fisher’s exact test for qualitative parameters

*EOI* early onset infection, *GA* gestational age, *IUGR* intrauterine growth restriction, *ANCS* antenatal corticosteroids: complete course including two doses of betamethasone given >24 h prior to birth, last dose <7 days before birth, *CRIB* critical risk index for babies, *RDS* respiratory distress syndrome, *IVH* intraventricular hemorrhage, *BPD* bronchopulmonary dysplasia, *ROP* retinopathy of prematurity, *LEU* leucocytes, *segNEU* segmented neutrophils, *bandNEU* band neutrophils, *juvNEU* juvenile neutrophils, *LYM* lymphocytes, *MON* monocytes

^a^Any ANCS before birth

^b^Value below lower determination threshold (4 mg/dl) was set to be 4 mg/dl

**Fig. 5:**
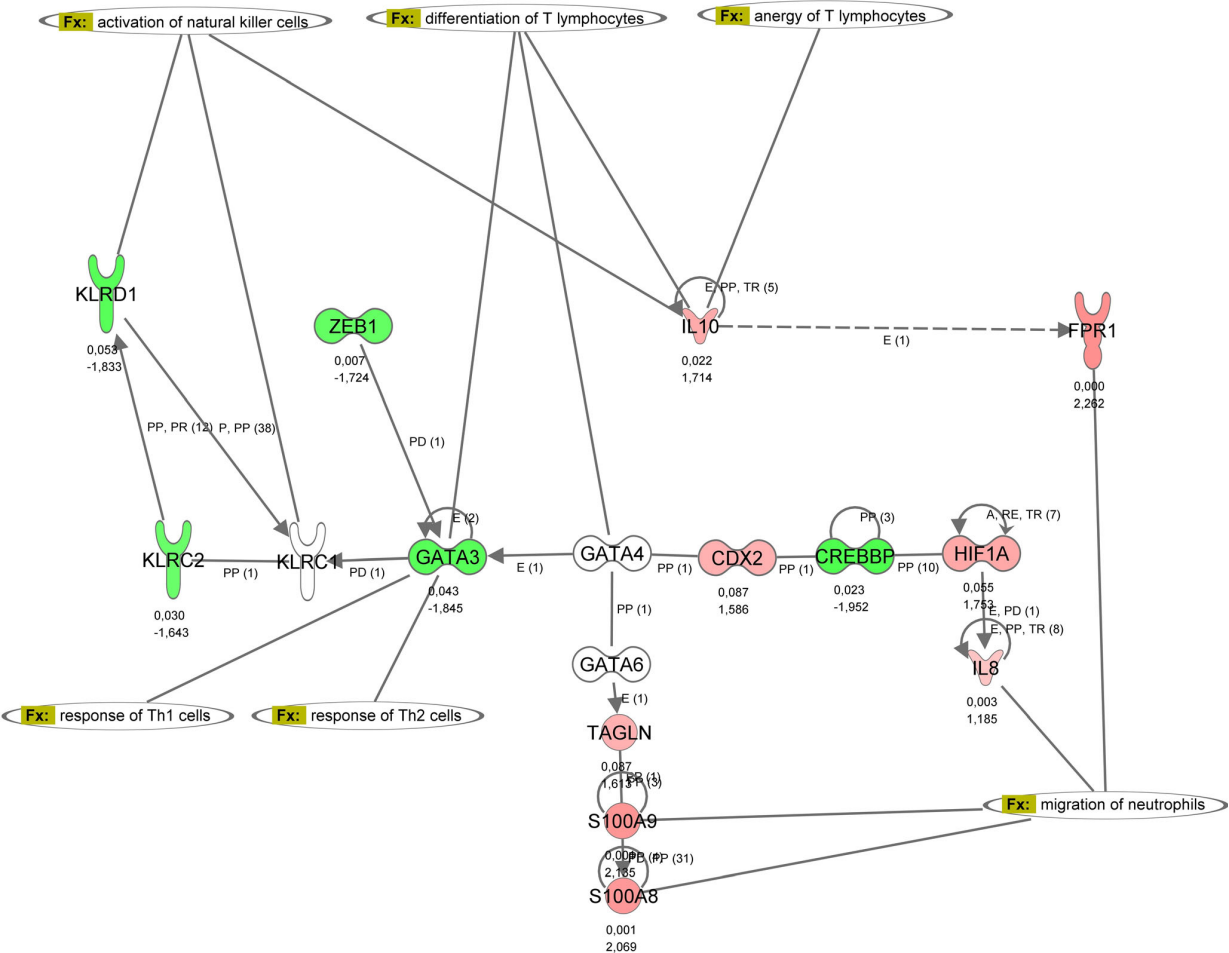
Influence of GATA3 and IL10 on regulation of NK cells and lymphocytes. The gene interaction network *influence of GATA3 and IL10 on regulation of NK cells and lymphocytes* of the differentially regulated genes in EOI shows the interaction of GATA3, IL10, and CREBBP with KLRs. Relationships and interactions between molecules are given in the figure legends of Fig. 4

Two transcription factors, GATA-binding protein 3 (GATA3) and CREB-binding protein (CREBBP), which are known to regulate the expression of KLRs were downregulated. GATA3 is a transcription factor preferentially expressed in NK and T cells that plays an important role in the early phase of NK cell development. Its activity is crucial for the diversification of the NK cell receptor repertoire and interferon γ (IFNG) production and thus pivotal for an effective NK cell response to viruses and bacteria. The downregulation of GATA3 and CREBBP in infants with EOI could explain the reduced gene expression of NK cell receptors in these patients as depicted in the interaction network in Fig. 5.

The measurement of NK cell counts in a cohort of preterm infants with and without EOI showed no significant difference in NK cell number between the two groups (non-EOI 7.8 ± 5.3 cells/μl vs. EOI 5.8 ± 5.3 cells/μl; mean and standard deviation (SD) each). This result suggested that the downregulation of NK cell activating genes as seen in EOI was not related to the NK cell count.

### Validation of microarray results

To validate the microarray data, TaqMan quantitative RT-PCR was performed on 10 human target genes involved in EOI (ANXA1, CD163, GNLY, HIF1A, KLRC2, KLRD1, MPO, PGLYRP1, TNFRSF10A, CD177). The overall correspondence between mRNA levels measured by microarrays and by RT-PCR analyses was high (*R* ^2^ = 0.88) (Supplemental Fig. 1).

### Replication of the results in an independent validation cohort

To test the reproducibility of the obtained results, 10 selected genes were analyzed by RT-PCR in a validation cohort (*n* = 43, Supplemental Table 2). The results show an overall good correlation (*R* ^2^ = 0.74) of the gene expression between the exploration and the validation cohort of preterm infants indicating reproducibility of the results (Supplemental Fig. 2).

**Fig. 6:**
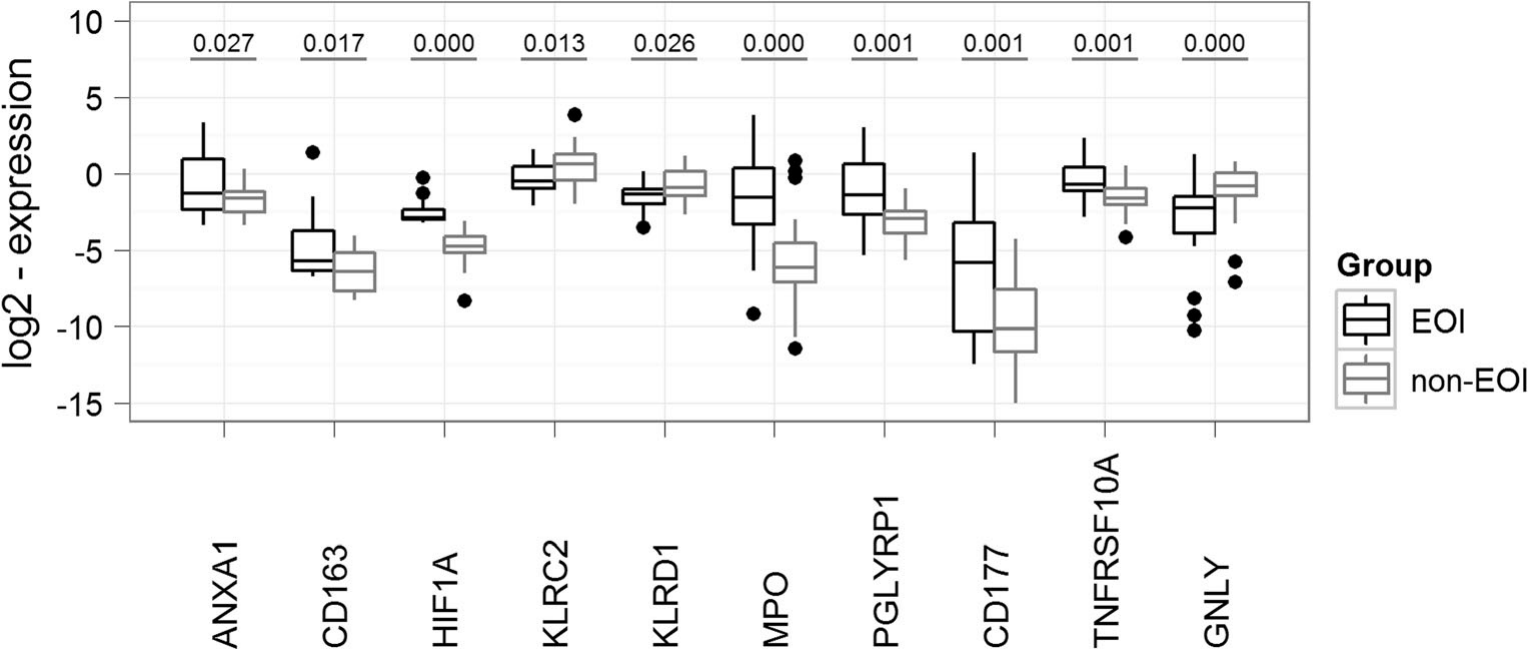
Comparison of gene expression between EOI and non-EOI for selected genes in a second patient cohort (replication cohort). TaqMan quantitative RT-PCR results of 10 selected genes were compared in infants with and without EOI within the replication cohort: RT-PCR results confirmed a significant overexpression of ANXA1, CD163, MPO, PGLYRP1, HIF1A, TNFRSF10A, and CD177 and a significant downregulation of genes involved in NK cell activation, i.e., KLRC2, KLRD1, and GNLY in the group of infants with EOI. *P* values are given for ANXA1, PGLYRP1, and TNFRSF10A using the Welch test, for CD163, HIF1A, and GNLY using the parametric Kruskal-Wallis rank-sum test, and for KLRC2, KLRD1, MPO, and CD177 using the Student’s *t* test

The RT-PCR results in the validation cohort confirmed significant upregulation of ANXA1, CD163, MPO, PGLYRP1, HIF1A, TNFRSF10A, and CD177 in the group of infants with EOI. In contrast, genes involved in NK cell activation, i.e., KLRC2, KLRD1, and GNLY, were found to be significantly downregulated (Fig. 6).

The results show that the validation cohort could support the key biologic findings found in the exploration cohort.

## Discussion and conclusions

We performed transcriptional profiling from umbilical arterial blood samples to obtain insights into the pathways involved in early EOI development in very premature infants. Comparison of the gene expression profiles of infants with EOI and without EOI revealed NK cell inactivation to be a hallmark of EOI discriminating EOI and non-EOI neonates (Figs. 1 and 2).

Impairment of NK cell function plays a critical role in the host response to infectious challenges in preterm infants with EOI, but a comprehensive evaluation of their cell state is lacking [22]. Our results indicate that decreased activation through downregulation of key surface markers, their regulating transcription factors GATA3 and CREBBP and downstream lytic enzymes account for the impairment of NK cell function, while NK cell numbers were similar among patients with and without EOI. NK cell interactions with other immune cells are regulating a wide range of immune responses [23] including bacterial clearance during bacterial sepsis by NK cell and macrophage interaction [24]. The decrease in NK cell activation may contribute to impaired clearance of pathogens leading to overwhelming systemic infections, frequent in premature infants. Insufficient elimination of pathogens due to impaired orchestration by NK cells could also explain the excessive neutrophil response in patients with EOI, consistent with findings from studies in infants with fetal inflammatory response syndrome (FIRS) [25].

Recent studies in infants up to 3 years of age revealed impaired adaptive immune system responses and specifically inhibition of NK cell activation in septic shock, supporting a key observation in our study [26, 27]. This is consistent with the findings of El-Sameea et al. [28] and Georgeson et al. [29], who showed a positive correlation between reduced NK cell activity and the presence, severity, and outcome of neonatal sepsis in term newborns.

Here, we show that similar concepts apply to EOI in preterm infants and validated the expression of key markers of NK cell activation (and the inhibition thereof) in an independent cohort. Overall, these findings suggest that a qualitative NK cell impairment, measured by gene expression profiling, may be inherent to EOI and provide a superior diagnostic tool for the early detection of EOI.

We identified two subclasses within the EOI cohort (EOI* and EOI**) characterized by increased neutrophil activation, T cell proliferation, hypoxia-induced signaling, and carbohydrate metabolism in EOI** preterm infants. Clinically, the group of infants classified as EOI* showed more infection-associated complications when compared with EOI**. In the light of impaired NK cell activation in both EOI subclasses, increased activation of neutrophils, T cell proliferation, hypoxia-induced signaling, and carbohydrate metabolism might indicate protective mechanisms helping to prevent complications of EOI in preterm infants when classified as EOI**.

To conclude, the results of this study indicate that (1) transcriptome patterns derived from umbilical arterial blood enable the discrimination of preterm infants with and without EOI and that (2) the group of preterm infants with EOI can be divided in two subclasses showing a common phenomenon of dysregulated NK cell activation but differing in the activation of neutrophils, T cell proliferation, hypoxia-induced signaling, and carbohydrate metabolism.

We propose that the addition of NK cell activity into the standard diagnostic repertoire for critically ill (preterm) neonates could be a useful complement to current laboratory diagnostics in order to improve early diagnosis of EOI.

## Electronic supplementary material

ESM 1 (PDF 464 kb)

## References

[1] Slattery MM, Morrison JJ (2002). Preterm delivery. Lancet.

[2] Stoll BJ, Gordon T, Korones SB, Shankaran S, Tyson JE, Bauer CR, Fanaroff AA, Lemons JA, Donovan EF, Oh W (1996). Early-onset sepsis in very low birth weight neonates: a report from the National Institute of Child Health and Human Development Neonatal Research Network. J Pediatr.

[3] Stoll BJ, Hansen NI, Sanchez PJ, Faix RG, Poindexter BB, Van Meurs KP, Bizzarro MJ, Goldberg RN, ID F, Hale EC (2011). Early onset neonatal sepsis: the burden of group B streptococcal and *E. coli* disease continues. Pediatrics.

[4] Jobe AH (2003). Antenatal factors and the development of bronchopulmonary dysplasia. Semin Neonatol.

[5] Leviton A, Kuban K, O’Shea TM, Paneth N, Fichorova R, Allred EN, Dammann O (2011). The relationship between early concentrations of 25 blood proteins and cerebral white matter injury in preterm newborns: the ELGAN study. J Pediatr.

[6] Laborada G, Rego M, Jain A, Guliano M, Stavola J, Ballabh P, Krauss AN, Auld PA, Nesin M (2003). Diagnostic value of cytokines and C-reactive protein in the first 24 hours of neonatal sepsis. Am J Perinatol.

[7] Mathers NJ, Pohlandt F (1987). Diagnostic audit of C-reactive protein in neonatal infection. Eur J Pediatr.

[8] Ottolini MC, Lundgren K, Mirkinson LJ, Cason S, Ottolini MG (2003). Utility of complete blood count and blood culture screening to diagnose neonatal sepsis in the asymptomatic at risk newborn. Pediatr Infect Dis J.

[9] Connell TG, Rele M, Cowley D, Buttery JP, Curtis N (2007). How reliable is a negative blood culture result? Volume of blood submitted for culture in routine practice in a children’s hospital. Pediatrics.

[10] Berger C, Uehlinger J, Ghelfi D, Blau N, Fanconi S (1995). Comparison of C-reactive protein and white blood cell count with differential in neonates at risk for septicaemia. Eur J Pediatr.

[11] Rodwell RL, Taylor KM, Tudehope DI, Gray PH (1993). Hematologic scoring system in early diagnosis of sepsis in neutropenic newborns. Pediatr Infect Dis J.

[12] Squire E, Favara B, Todd J (1979). Diagnosis of neonatal bacterial infection: hematologic and pathologic findings in fatal and nonfatal cases. Pediatrics.

[13] Xanthou M (1970). Leucocyte blood picture in healthy full-term and premature babies during neonatal period. Arch Dis Child.

[14] Xanthou M (1972). Leucocyte blood picture in ill newborn babies. Arch Dis Child.

[15] Gerdes JS (1991). Clinicopathologic approach to the diagnosis of neonatal sepsis. Clin Perinatol.

[16] Josse J, Husson F (2013). Handling missing values in exploratory multivariate data analysis methods. Journal de la Société Française de Statistique.

[17] Bolstad BM, Irizarry RA, Astrand M, Speed TP (2003). A comparison of normalization methods for high density oligonucleotide array data based on variance and bias. Bioinformatics.

[18] Breitling R, Armengaud P, Amtmann A, Herzyk P (2004). Rank products: a simple, yet powerful, new method to detect differentially regulated genes in replicated microarray experiments. FEBS Lett.

[19] Leek JT, Storey JD (2007). Capturing heterogeneity in gene expression studies by surrogate variable analysis. PLoS Genet.

[20] Daffertshofer A, Lamoth CJ, Meijer OG, Beek PJ (2004). PCA in studying coordination and variability: a tutorial. Clin Biomech (Bristol, Avon).

[21] Porter JD, Merriam AP, Leahy P, Gong B, Khanna S (2003). Dissection of temporal gene expression signatures of affected and spared muscle groups in dystrophin-deficient (mdx) mice. Hum Mol Genet.

[22] Marodi L (2006). Neonatal innate immunity to infectious agents. Infect Immun.

[23] Zhang C, Zhang J, Tian Z (2006). The regulatory effect of natural killer cells: do "NK-reg cells" exist?. Cell Mol Immunol.

[24] Scott MJ, Hoth JJ, Gardner SA, Peyton JC, Cheadle WG (2003). Natural killer cell activation primes macrophages to clear bacterial infection. Am Surg.

[25] Madsen-Bouterse SA, Romero R, Tarca AL, Kusanovic JP, Espinoza J, Kim CJ, Kim JS, Edwin SS, Gomez R, Draghici S (2010). The transcriptome of the fetal inflammatory response syndrome. Am J Reprod Immunol.

[26] Adkins B, Leclerc C, Marshall-Clarke S (2004). Neonatal adaptive immunity comes of age. Nat Rev Immunol.

[27] Ridge JP, Fuchs EJ, Matzinger P (1996). Neonatal tolerance revisited: turning on newborn T cells with dendritic cells. Science.

[28] El-Sameea ER, Metwally SS, Mashhour E, El-Bendary A, Hassan AM, El-Sharkawy H, El-Shennawy FA (2004). Evaluation of natural killer cells as diagnostic markers of early onset neonatal sepsis: comparison with C-reactive protein and interleukin-8. Egypt J Immunol.

[29] Georgeson GD, Szony BJ, Streitman K, Kovacs A, Kovacs L, Laszlo A (2001). Natural killer cell cytotoxicity is deficient in newborns with sepsis and recurrent infections. Eur J Pediatr.

