# Supplementary material for: Gene expression profiling at birth characterizing the preterm infant with early onset infection
Source: J Mol Med (Berl). 2016 Aug 30;95(2):169–80. doi: 10.1007/s00109-016-1466-4 (PMC5239802; doi:10.1007/s00109-016-1466-4)
Supplement: Supplementary file 1 — (PDF 464 kb) [file 109_2016_1466_MOESM1_ESM.pdf]

Electronic supplement

Gene expression profiling at birth characterizing the preterm infant with early onset Infection – Hilgendorff et al – J Mol Med 2016

SUPPLEMENTAL MATERIALS AND METHODS

Gene expression profiling

Twenty four patients were included in the gene expression analysis study. The PAXgene Blood RNA System (PreAnalytiX, Heidelberg, Germany) was used to collect umbilical arterial blood from an indwelling umbilical artery catheter and isolate the RNA according to the manufacturer’s recommendations (PreAnalytiX). Total RNA was quantified with Nanodrop (NanoDrop Technologies, Rockland DE, USA) and the quality of RNA was assessed using the Agilent 2100 Bioanalyzer Bioanalyzer (Agilent Technologies GmbH, Boeblingen, Germany). When the total RNA fulfilled quality criteria such as sufficient yield (> 2 µg), a 260/280-ratio of > 1.9 and electrophoretic profiles showing clear and sharp ribosomal peaks, the RNA was subjected to cRNA synthesis, cRNA fragmentation and finally hybridization on CodeLink UniSet Human 10 K Bioarrays (GE Healthcare, Freiburg, Germany) using the CodeLink Expression Assay Kit (GE Healthcare) according to the manufacturer's instructions. Each patient sample was hybridized on at least two bioarrays (technical replicates). Bioarrays were stained with Cy5™-streptavadin (GE Healthcare) and scanned using the GenePix® 4000 B scanner and the GenePix Pro 4.0 Software (Axon Instruments, Arlington, USA). A total of 75 array images were subjected to data analysis.

Spot signals of CodeLink bioarrays were quantified using CodeLink Expression Software V1.21 (GE Healthcare), as outlined in the user's manual. CodeLink Expression Software V4.1 generated background corrected raw data as well as median- centered intra-slide normalized data. The intra-slide normalized data were used for further analysis. The software automatically calculated thresholds for intra-slide normalized intensities for each array and flagged genes as TRUE when the gene intensity was higher than the threshold or FALSE when the intensity was lower than the threshold. The present call of a microarray was given as the ratio of genes flagged as TRUE / total number of genes on microarray. Microarrays subjected to data analysis showed a mean present call of 81%, indicating a high number of genes above threshold, i.e. being flagged as TRUE. Furthermore, the software

flagged each gene value as GOOD, EMPTY, POOR, NEG or MSR, thus defining different quality measures as outlined in the user's manual. Only gene values flagged as GOOD or EMPTY were used in the following analysis workflow:

1) Definition of patient groups:

Preterm infants and corresponding microarrays were separated in into two groups (dataset 1):

Group 1: 8 infants (NS1-8; 19 microarrays) without EOI

Group 2: 16 infants (EOI1-16; 44 microarrays) with EOI

2) Removal of genes with a high number of missing values or of values flagged as FALSE:

Genes with missing values in ≥ 50% of all arrays in a group were excluded from the dataset. Genes that were flagged as FALSE in > 50% of arrays in each group were also excluded from the dataset.

3) Imputation of remaining missing values:

Remaining missing values were imputed using sequential K-nearest neighbour (SKNN) imputation with k=5 [9].

4) Normalization of imputed dataset:

Imputed dataset was normalized using quantiles normalization in R and logged to base 2 [2].

5) Array outlier detection:

Dissimilarity matrices of the normalized dataset were generated in AVADIS-Pride to determine outlier arrays within the dataset. No outlier arrays were identified in the data set [7].

6) Statistical analysis of microarrays:

For each gene, the mean value of all technical replicates of an infant was calculated in dChip (4). To identify differentially regulated genes between group 1 (NS) and group 2 (EOI), the dataset was subjected to a two-class rank statistics (Rank products, RP) as described below (5, 6). For each gene, a false discovery rate (FDR) ≤ 0.1 was defined as the significance level.

7) Annotation of genes:

Significantly regulated genes were annotated using the web-based annotation tools SOURCE [6] and the Database for Annotation, Visualization and Integrated Discovery (DAVID Bioinformatics Resources 2008) [5] as described in the manual.

8) Enriched functional categories:

Enriched functional categories within the differentially regulated genes were determined using DAVID [5] version 6.7. DAVID is a platform that provides statistical methods (reported as an Enrichment Score or EASE score) to facilitate the biological interpretation of gene lists deriving from microarray

analysis. Enriched genes describe a class of genes that have similar functions regardless of their expression level. They appear in a list of interest more often than what would normally be predicted by their distribution among all genes assayed. An EASE score is calculated for likelihood of enrichment of biological processes, molecular functions and cellular component categories using the Gene Ontology (GO) public database. It is derived from an adaption of the Fisher's Exact test. The Enrichment Score for a cluster of similar terms is calculated by using the negative logarithm of the geometrical mean of all EASE scores of the cluster. Comparison of functional annotation analysis results was conducted using DAVID overrepresentation analysis of Biological Process Gene Ontology terms, so called GO BP fat terms, and KEGG pathways with an EASE score of at least 10%.

9) Cluster analysis:

Hierarchical cluster analysis of the significantly regulated genes (FDR ≤ 0.1) was performed using the centroid linkage method and the distance matrix 1 –r in dChip [13].

10) Network analysis:

Identification of networks in the differentially regulated genes was achieved using Ingenuity Pathways Analysis (Ingenuity® Systems, www.ingenuity.com)

Network Generation:

These genes were overlaid onto global molecular networks contained in the Ingenuity Pathways Knowledge Base. Networks of the genes were then algorithmically generated based on their connectivity.

Functional Analysis and network generation:

A data set containing gene identifiers and corresponding expression values was uploaded into the application. Each gene identifier was mapped to its corresponding gene object in the Ingenuity Pathways Knowledge Base. An FDR ≤ 0.1 was set to identify genes whose expression was significantly differentially regulated. The Functional Analysis identified the biological processes that were most significant to the data set. Only genes that met the criteria FDR ≤ 0.1 were assigned to biological processes using the Ingenuity Pathways Knowledge Base. The probability for each biological process was given as a p-value determined by the Fisher's exact test.

Surrogate Variable Analysis (SVA)

SVA and limma were used to account for potential confounding effects as well as further hidden structures.

SVA [12] analysis was conducted according to Leek et al.'s R-package description (Version 3.12.0) [11] and estimated two statistical models: The first model accounted for the confounders identified to correlate with structural differences between the study groups (EOI and non-EOI), i.e. GA, birthweight, and WBC (leucocytes (LEU), segmented neutrophils (segNEU), band neutrophils (bandNEU), juvenile neutrophils, lymphocytes (LYM) and monocytes (MON)). The second model accounted for confounders as well as EOI status, thereby protecting the EOI status. Gene expression data were then fitted using Limma (linear models for microarray analysis) implementing the calculated surrogate variable (SVA) together with the confounding variables in the final statistical analysis [14, 15]. The number of surrogate variables used for gene expression adjustment was estimated using Leek's asymptotic conditional singular value decomposition [10].

Limma, a linear model is used to explain the variance of every gene or transcript by the specified predictors. As gene expressions in a microarray experiment are not independent from each other, a hierarchical model is defined best describing the variance for the coefficients and expressions across genes. Prior distributions for the coefficient and expression variances are estimated using an empirical Bayes approach taking the inter-dependency of gene expression into account. Posterior values are obtained by adjusting prior values to the actual observed values [14]. Subsequently, adjusted gene expression data were obtained as residuals of the limma-model and used to calculate Rank Products for EOI status.

Transcripts were considered as statistically significantly associated with either WBC or EOI if FDR ≤ 0.1.

Rank products

The Rank Products method [3, 4]was used for identifying differentially expressed genes in the expression data. The method is based on the premise that a gene in an experiment examining *n* genes in *k* replicates, has a probability of being ranked first (rank 1) of 1/*nk* if the lists were entirely random. Therefore, it is unlikely for a single gene to be in the top position in all replicates if this gene was not differentially expressed, i.e., if all null hypotheses were true. More generally, for each gene *g* in *k* replicates *i*, each examining *ni* genes, one can calculate the corresponding combined probability as a rank product $RP_g^{up}=\prod_{i=1}^{k}(r_{i,g}^{up/ni})$ where $r_{i,g}$ up is the position of gene *g* in the list of genes in the *i*th replicate sorted by decreasing fold change, i.e. r^{up} = 1 for the most strongly upregulated gene, etc. The genes can then be sorted according to the likelihood of observing their RP value at or above a certain

position on the list. Analogously, RP^{down}_{g} is calculated from the list of genes sorted by increasing FC, i.e. r^{down}=1 for the most strongly down-regulated gene.

To know how significant the changes are and how many of the selected genes are likely to be truly differentially expressed, a simple permutation-based estimation procedure provides a very convenient way to determine how likely it is to observe a given RP value in a random experiment by converting the RP value to an E value in analogy to the BLAST results [1]. The RP value distribution can be approximated in each case by calculating the RP values for a number of *z* random "experiments" with the same number of replicates and "genes" as the real experiment. Each random experiment consists of *k* random permutations of the numbers 1,…,*n* and for these the RP values are calculated as described above. The number of simulated RP values in the random experiments that are smaller than or equal to a given experimental RP value (x(RP)) are then used to calculate the average expected value $E(\mathrm{RP}) \approx x(\mathrm{RP})/z$.

Subsequently, for each gene g a conservative estimate of the percentage of false-positives (PFP) is calculated: $q_g = E(\mathrm{RP}_g)/\mathrm{rank}(_g)$. Here, rank($g$) denotes the position of gene g in a list of all genes sorted by increasing RP value, i.e., it is the number of genes accepted as significantly regulated. This estimates the false discovery rate (FDR) [16] and provides a flexible way to assign a significance level to each gene. The FDR is accepted as a reasonable significance threshold in microarray studies [16]. One can now decide how large a PFP would be acceptable and extend the list of accepted genes up to the gene with this q_g value. The rank product method was chosen since it has been shown to outperform classical t-statistic and moderated t-statistics when datasets have low numbers of samples or high levels of noise [3, 8].

RT-PCR

Real time RT-PCR was performed for 10 human target genes deriving from the microarray results (ANXA1, CD163, GNLY, HIF1A, KLRC2, KLRD1, MPO, PGLYRP1, TNFRSF10A, CD177) and three housekeeping genes G6PD (glucose-6-phosphate dehydrogenase), SDHA (succinate dehydrogenase complex, subunit A, flavoprotein (Fp)) and PGK1 (phosphoglycerate kinase 1) .

QuantiTect Primer Assays for selected genes were obtained from the Gene Globe Portal (Qiagen, Hilden, Germany). Prior to sample measurements, all primer pairs were validated using a control total RNA pool derived from PAXgene samples. Standard curves of gradual RNA dilutions were designed by plotting Ct values against the log-transformed input total RNA (in ng). Amplification efficiencies for

the target genes and the internal controls were calculated as E = 10(-1/S) -1, where S is the slope of the standard curve. The amplification efficiencies are given in Supp. Table 3. For sample measurements, 400 ng PAXgene RNA of 8 non-EOI and 16 EOI samples were subjected to cDNA synthesis using SuperScript II (Invitrogen) and a mixture of T21 and random nonamer primers (Metabion) following the instructions for the reverse transcription reaction recommended for the QuantiTect SYBR Green Kit (Qiagen). Real-time RT-PCR was performed on the ABI PRISM® 7900 Sequence Detection System (Applied Biosystems, Darmstadt, Germany) using the Quantitect SYBR Green PCR Kit (Qiagen) with cDNA corresponding to 2 ng (0,5%) input total RNA. All reactions were run in duplicate. Ct values of the tested genes were determined and compared with the respective standard curve. The antilogarithm of the value at the intersection point with the standard curve corresponded with the amount of human total RNA of the expressed target gene. The normalized expression of a target gene Eg was given as the ratio between the total RNA amount of the target gene and the mean of the three internal controls G6PD, SDHA and PGK1. Both normalized microarray intensities and RT gene expression levels relative to internal controls of infants with EOI were log2 transformed and expressed as log2 differences from patients without EOI.

REFERENCES

1. Altschul SF, Gish W, Miller W, Myers EW, Lipman DJ (1990) Basic local alignment search tool. J Mol Biol 215:403–410. doi: 10.1016/S0022-2836(05)80360-2

2. Bolstad BM, Irizarry RA, Åstrand M, Speed TP (2003) A comparison of normalization methods for high density oligonucleotide array data based on variance and bias. Bioinformatics 19:185–193. doi: 10.1093/bioinformatics/19.2.185

3. Breitling R, Armengaud P, Amtmann A, Herzyk P (2004) Rank products: a simple, yet powerful, new method to detect differentially regulated genes in replicated microarray experiments. FEBS Lett 573:83–92. doi: 10.1016/j.febslet.2004.07.055

4. Breitling R, Herzyk P (2005) Rank-based methods as a non-parametric alternative of the T-statistic for the analysis of biological microarray data. J Bioinform Comput Biol 3:1171–1189.

5. Dennis G, Sherman BT, Hosack DA, Yang J, Gao W, Lane HC, Lempicki RA (2003) DAVID: Database for Annotation, Visualization, and Integrated Discovery. Genome Biol 4:R60. doi: 10.1186/gb-2003-4-9-r60

6. Diehn M, Sherlock G, Binkley G, Jin H, Matese JC, Hernandez-Boussard T, Rees CA, Cherry JM, Botstein D, Brown PO, Alizadeh AA (2003) SOURCE: a unified genomic resource of functional annotations, ontologies, and gene expression data. Nucleic Acids Res 31:219–223. doi: 10.1093/nar/gkg014

7. Gwadry FG, Sequeira A, Hoke G, Ffrench-Mullen JMH, Turecki G (2005) Molecular characterization of suicide by microarray analysis. Am J Med Genet C Semin Med Genet 133C:48–56. doi: 10.1002/ajmg.c.30046

8. Jeffery IB, Higgins DG, Culhane AC (2006) Comparison and evaluation of methods for generating differentially expressed gene lists from microarray data. BMC Bioinformatics 7:359. doi: 10.1186/1471-2105-7-359

9. Kim K-Y, Kim B-J, Yi G-S (2004) Reuse of imputed data in microarray analysis increases imputation efficiency. BMC Bioinformatics 5:160. doi: 10.1186/1471-2105-5-160

10. Leek JT (2011) Asymptotic conditional singular value decomposition for high-dimensional genomic data. Biometrics 67:344–352. doi: 10.1111/j.1541-0420.2010.01455.x

11. Leek JT, Johnson WE, Parker HS, Fertig EJ, Jaffe AE, Storey JD (2014) sva: Surrogate Variable Analysis. http://bioconductor.org/packages/sva/.

12. Leek JT, Storey JD (2007) Capturing Heterogeneity in Gene Expression Studies by Surrogate Variable Analysis. PLOS Genet 3:e161. doi: 10.1371/journal.pgen.0030161

13. Li C, Wong WH (2001) Model-based analysis of oligonucleotide arrays: expression index computation and outlier detection. Proc Natl Acad Sci U S A 98:31–36. doi: 10.1073/pnas.011404098

14. Smyth GK (2004) Linear models and empirical Bayes methods for assessing differential expression in microarray experiments. Stat Appl Genet Mol Biol 3:1–25. doi: 10.2202/1544-6115.1027

15. Smyth GK (2005) Limma: linear models for microarray data. In: Gentleman R, Carey V, Dudoit S, Irizarry R, Huber W (eds) Bioinforma. Comput. Biol. Solut. Using R Bioconductor. Springer, New York, pp 397–420

16. Storey JD, Tibshirani R (2003) Statistical significance for genomewide studies. Proc Natl Acad Sci U S A 100:9440–9445. doi: 10.1073/pnas.1530509100

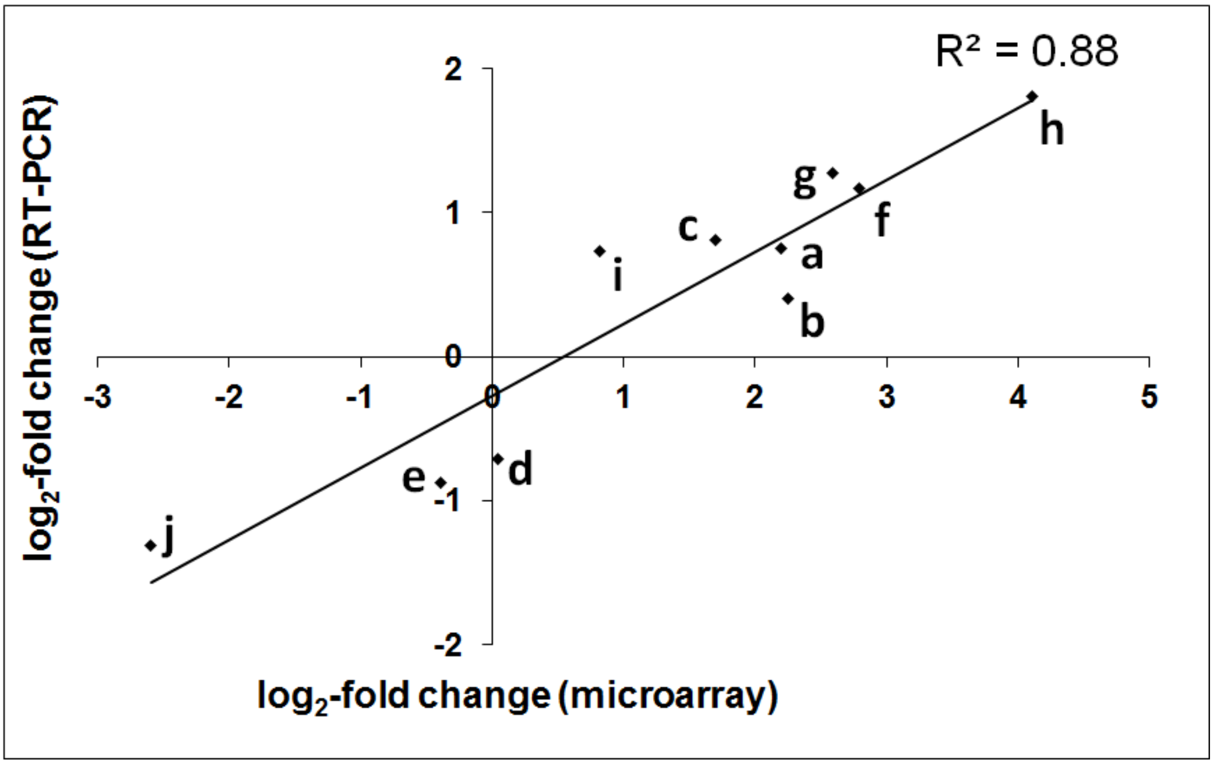

Suppl. Figure 1: Validation of microarray results by real-time RT-PCR.

Validation of microarray results by TaqMan quantitative real-time PCR (RT-PCR) of selected genes: a: ANXA1; b: CD163; c: HIF1A; d: KLRC2; e: KLRD1; f: MPO; g: PGLYRP1; h: CD177; i: TNFRSF10A; j: GNLY. A strong correlation between the log2-fold changes deriving from microarrays and from RT-PCR is mirrored by the coefficient of determination $R^2 = 0.88$ and the coefficient of correlation $r = 0.94$.

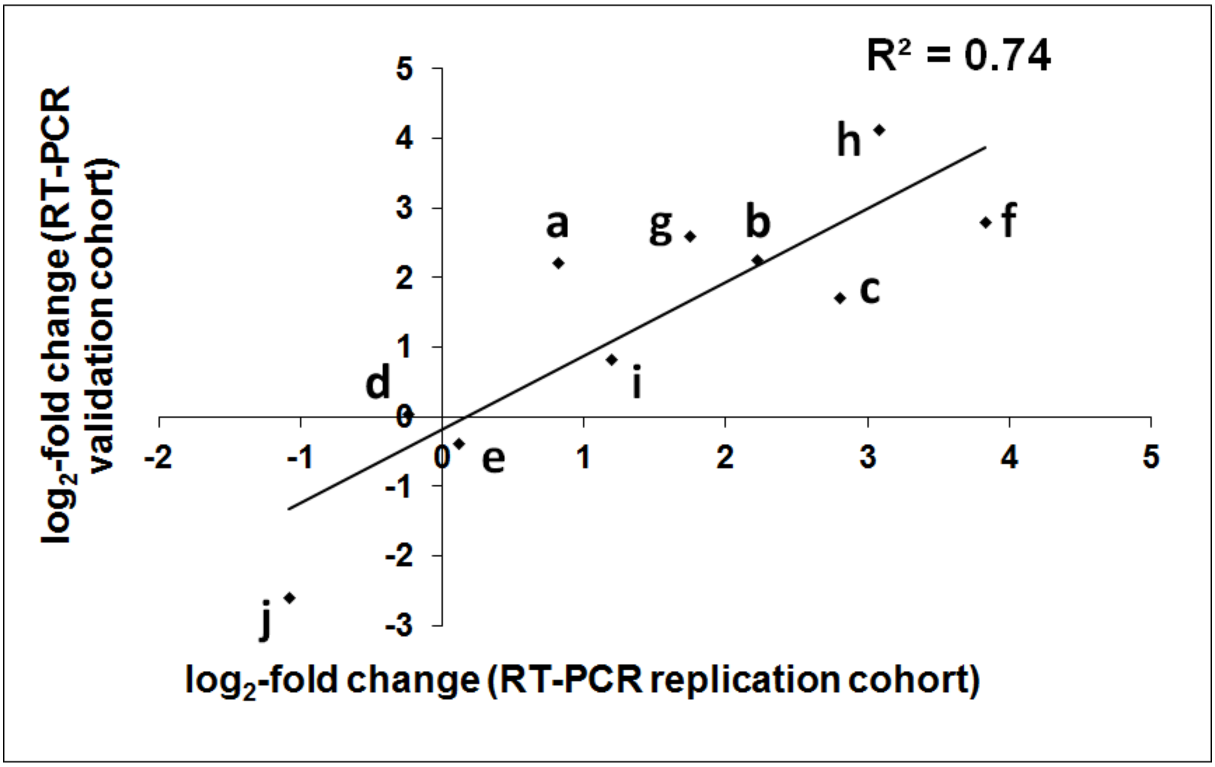

Suppl. Figure 2: Replication of the microarray results in a second patient cohort

For replication of the microarray results, RT-PCR results obtained from the cohort initially tested with microarrays were correlated with RT-PCR results obtained from an independent patient cohort. TaqMan quantitative RT-PCR were performed for selected genes: a: ANXA1; b: CD163; c: HIF1A; d: KLRC2; e: KLRD1; f: MPO; g: PGLYRP1; h: CD177; i: TNFRSF10A; j: GNLY. An overall good correlation between the log2-fold changes deriving from the initial microarray cohort and from the independent replication cohort is mirrored by the coefficient of determination $R^2 = 0.74$ and the coefficient of correlation $r = 0.86$.

Supplemental Table 1A (up regulated genes, FDR < 0.1)

GenBank Acc.	Gene Name	Gene Symbol	Fold change	FDR
AF090101	AP2 associated kinase 1	AAK1	1.56	0.075
NM_022977	Acyl-CoA synthetase long-chain family member 4	ACSL4	1.90	0.016
AB020644	Acyl-CoA synthetase long-chain family member 6	ACSL6	1.98	0.000
NM_003816	ADAM metallopeptidase domain 9	ADAM9	1.55	0.035
NM_001124	Adrenomedullin	ADM	2.27	0.000
NM_017657	Aftiphilin	AFTPH	2.00	0.019
NM_004504	ArfGAP with FG repeats 1	AGFG1	2.08	0.010
NM_022831	Axin interactor, dorsalization associated	AIDA	1.82	0.079
NM_000689	Aldehyde dehydrogenase 1 family, member A1	ALDH1A1	1.73	0.005
NM_001141	Arachidonate 15-lipoxygenase, type B	ALOX15B	1.52	0.086
NM_000478	Alkaline phosphatase, liver/bone/kidney	ALPL	2.32	0.000
NM_000700	Annexin A1	ANXA1	1.69	0.042
NM_005139	Annexin A3	ANXA3	2.60	0.000
NM_020980	Aquaporin 9	AQP9	1.76	0.008
NM_001657	Amphiregulin	AREG	2.23	0.000
NM_012106	ADP-ribosylation factor-like 2 binding protein	ARL2BP	1.91	0.033
NM_015396	Armadillo repeat containing 8	ARMC8	1.96	0.021
NM_024095	Ankyrin repeat and SOCS box-containing 8	ASB8	1.93	0.021
NM_032204	Activating signal cointegrator 1 complex subunit 2	ASCC2	2.02	0.020
NM_032466	Aspartate beta-hydroxylase	ASPH	1.39	0.069
NM_004046	ATP synthase, H+ transporting, mitochondrial F1 complex, alpha subunit 1, cardiac muscle	ATP5A1	1.62	0.092
NM_013236	Ataxin 10	ATXN10	2.00	0.014
NM_001700	Azurocidin 1	AZU1	1.57	0.001
NM_013448	Bromodomain adjacent to zinc finger domain, 1A	BAZ1A	1.89	0.051
NM_004049	BCL2-related protein A1	BCL2A1	1.83	0.004
NM_015367	BCL2-like 13 (apoptosis facilitator)	BCL2L13	2.66	0.000
NM_001706	B-cell CLL/lymphoma 6	BCL6	2.00	0.017
NM_001166	Baculoviral IAP repeat-containing 2	BIRC2	2.12	0.014
NM_004052	BCL2/adenovirus E1B 19kDa interacting protein 3	BNIP3	1.06	0.086
AL132665	BCL2/adenovirus E1B 19kDa interacting protein 3-like	BNIP3L	1.91	0.057
NM_014299	Bromodomain containing 4	BRD4	1.78	0.019
NM_020375	Chromosome 12 open reading frame 5	C12orf5	1.67	0.045

NM_018356	Chromosome 5 open reading frame 22	C5orf22	1.95	0.027
NM_000717	Carbonic anhydrase IV	CA4	1.87	0.027
NM_001747	Capping protein (actin filament), gelsolin-like	CAPG	1.53	0.039
NM_001752	Catalase	CAT	3.51	0.000
NM_001762	Chaperonin containing TCP1, subunit 6A (zeta 1)	CCT6A	1.89	0.047
NM_004244	CD163 molecule	CD163	1.33	0.059
NM_020406	CD177 molecule	CD177	3.49	0.000
NM_012072	CD93 molecule	CD93	1.61	0.022
NM_001785	Cytidine deaminase	CDA	1.90	0.004
NM_003903	Cell division cycle 16 homolog (S. cerevisiae)	CDC16	2.36	0.001
U51096	Caudal type homeobox 2	CDX2	1.59	0.087
X16354	Carcinoembryonic antigen-related cell adhesion molecule 1 (biliary glycoprotein)	CEACAM1	1.78	0.032
NM_004364	CCAAT/enhancer binding protein (C/EBP), alpha	CEBPA	2.10	0.002
NM_005194	CCAAT/enhancer binding protein (C/EBP), beta	CEBPB	2.35	0.000
NM_001270	Chromodomain helicase DNA binding protein 1	CHD1	1.42	0.094
NM_014358	C-type lectin domain family 4, member E	CLEC4E	1.34	0.050
NM_004368	Calponin 2	CNN2	2.27	0.004
NM_014900	COBL-like 1	COBLL1	1.50	0.098
NM_004766	Coatomer protein complex, subunit beta 2 (beta prime)	COPB2	2.23	0.003
NM_003915	Copine I	CPNE1	1.79	0.025
NM_013291	Cleavage and polyadenylation specific factor 1, 160kDa	CPSF1	1.78	0.075
NM_001316	CSE1 chromosome segregation 1-like (yeast)	CSE1L	2.14	0.007
NM_004078	Cysteine and glycine-rich protein 1	CSRP1	1.91	0.025
NM_005213	Cystatin A (stefin A)	CSTA	1.39	0.043
NM_003798	Catenin (cadherin-associated protein), alpha-like 1	CTNNAL1	1.76	0.021
NM_001909	Cathepsin D	CTSD	2.00	0.007
NM_004079	Cathepsin S	CTSS	1.76	0.019
NM_003467	Chemokine (C-X-C motif) receptor 4	CXCR4	1.69	0.022
NM_000104	Cytochrome P450, family 1, subfamily B, polypeptide 1	CYP1B1	1.48	0.026
AB002379	Dishevelled associated activator of morphogenesis 2	DAAM2	1.72	0.025
AB067479	DDB1 and CUL4 associated factor 12	DCAF12	2.33	0.002
NM_006400	Dynactin 2 (p50)	DCTN2	1.70	0.087
NM_001923	Damage-specific DNA binding protein 1, 127kDa	DDB1	2.05	0.006
NM_003676	Degenerative spermatocyte homolog 1, lipid desaturase (Drosophila)	DEGS1	1.84	0.082
AL079292	DEAH (Asp-Glu-Ala-His) box polypeptide 29	DHX29	1.95	0.024

NM_000108	Dihydrolipoamide dehydrogenase	DLD	2.36	0.001
NM_016364	Dual specificity phosphatase 13	DUSP13	1.74	0.051
AK055491	Dynein, cytoplasmic 1, intermediate chain 2	DYNC1I2	1.88	0.059
NM_003752	Eukaryotic translation initiation factor 3, subunit C	EIF3C	3.71	0.000
NM_001418	Eukaryotic translation initiation factor 4 gamma, 2	EIF4G2	2.70	0.000
AF112219	Esterase D	ESD	2.21	0.002
NM_019018	Family with sequence similarity 105, member A	FAM105A	1.48	0.069
AL050100	Family with sequence similarity 119, member B	FAM119B	2.49	0.001
NM_016613	Family with sequence similarity 198, member B	FAM198B	1.74	0.021
AB011164	Family with sequence similarity 21, member C	FAM21C	1.60	0.056
NM_000566	Fc fragment of IgG, high affinity Ia, receptor (CD64)	FCGR1A	2.10	0.000
BC004988	Fem-1 homolog a (C. elegans)	FEM1A	1.76	0.086
NM_001456	Filamin A, alpha	FLNA	1.47	0.061
NM_004475	Flotillin 2	FLOT2	1.74	0.087
NM_002029	Formyl peptide receptor 1	FPR1	2.26	0.000
NM_000147	Fucosidase, alpha-L- 1, tissue	FUCA1	1.36	0.065
NM_022003	FXYD domain containing ion transport regulator 6	FXYD6	1.53	0.086
NM_004120	Guanylate binding protein 2, interferon-inducible	GBP2	1.81	0.065
NM_012198	Grancalcin, EF-hand calcium binding protein	GCA	2.79	0.000
NM_004125	Guanine nucleotide binding protein (G protein), gamma 10	GNG10	1.83	0.006
NM_032292	Gon-4-like (C. elegans)	GON4L	1.92	0.037
NM_000175	Glucose-6-phosphate isomerase	GPI	2.42	0.001
NM_005291	G protein-coupled receptor 17	GPR17	1.55	0.038
NM_004951	G protein-coupled receptor 183	GPR183	1.25	0.080
NM_003608	G protein-coupled receptor 65	GPR65	1.51	0.079
NM_004127	G protein pathway suppressor 1	GPS1	2.68	0.000
NM_004130	Glycogenin 1	GYG1	3.29	0.000
NM_005335	Hematopoietic cell-specific Lyn substrate 1	HCLS1	2.21	0.002
NM_015987	Heme binding protein 1	HEBP1	3.13	0.000
NM_032558	Hippocampus abundant transcript-like 1	HIATL1	1.70	0.045
NM_001530	Hypoxia inducible factor 1, alpha subunit (basic helix-loop-helix transcription factor)	HIF1A	1.75	0.055
NM_000188	Hexokinase 1	HK1	2.43	0.000
NM_000860	Hydroxyprostaglandin dehydrogenase 15-(NAD)	HPGD	1.67	0.065
NM_020995	Haptoglobin-related protein	HPR	4.26	0.000
NM_005348	Heat shock protein 90kDa alpha (cytosolic), class A member 1	HSP90AA1	1.79	0.057

NM_007355	Heat shock protein 90kDa alpha (cytosolic), class B member 1	HSP90AB1	2.31	0.002
NM_024012	5-hydroxytryptamine (serotonin) receptor 5A	HTR5A	1.66	0.025
NM_005534	Interferon gamma receptor 2 (interferon gamma transducer 1)	IFNGR2	1.88	0.004
NM_000572	Interleukin 10	IL10	1.71	0.022
NM_000584	Interleukin 8	IL8	1.19	0.003
AL049435	Interleukin-1 receptor-associated kinase 3	IRAK3	1.57	0.062
NM_002211	Integrin, beta 1 (fibronectin receptor, beta polypeptide, antigen CD29 includes MDF2, MSK12)	ITGB1	1.97	0.045
D79998	Potassium channel tetramerisation domain containing 2	KCTD2	1.78	0.053
AB029032	KIAA1109	KIAA1109	1.96	0.009
AK002186	Kruppel-like factor 11	KLF11	1.66	0.064
NM_003937	Kynureninase (L-kynurenine hydrolase)	KYNU	1.48	0.066
NM_005566	Lactate dehydrogenase A	LDHA	1.95	0.018
NM_006864	Leukocyte immunoglobulin-like receptor, subfamily B (with TM and ITIM domains), member 3	LILRB3	1.63	0.047
NM_016733	LIM domain kinase 2	LIMK2	1.78	0.011
NM_002318	Lysyl oxidase-like 2	LOXL2	2.04	0.002
NM_005767	Lysophosphatidic acid receptor 6	LPAR6	1.71	0.021
NM_052972	Leucine-rich alpha-2-glycoprotein 1	LRG1	1.80	0.032
NM_014045	Low density lipoprotein receptor-related protein 10	LRP10	2.46	0.000
AA868493	Leukotriene A4 hydrolase	LTA4H	2.34	0.003
NM_000895	Leukotriene A4 hydrolase	LTA4H	1.96	0.051
NM_002343	Lactotransferrin	LTF	2.82	0.000
NM_002350	V-yes-1 Yamaguchi sarcoma viral related oncogene homolog	LYN	1.66	0.068
AF055376	V-maf musculoaponeurotic fibrosarcoma oncogene homolog (avian)	MAF	1.95	0.014
S82470	Membrane bound O-acyltransferase domain containing 7	MBOAT7	1.62	0.035
NM_018349	Multiple C2 domains, transmembrane 2	MCTP2	1.55	0.045
NM_005481	Mediator complex subunit 16	MED16	2.02	0.002
AF084943	Multiple inositol-polyphosphate phosphatase 1	MINPP1	1.51	0.073
NM_017947	Molybdenum cofactor sulfurase	MOCOS	2.72	0.000
NM_000250	Myeloperoxidase	MPO	2.24	0.000
NM_012219	Muscle RAS oncogene homolog	MRAS	1.57	0.035
NM_006138	Membrane-spanning 4-domains, subfamily A, member 3 (hematopoietic cell-specific)	MS4A3	2.34	0.000
NM_024021	Membrane-spanning 4-domains, subfamily A, member 4	MS4A4A	1.91	0.005
AL162068	Nucleosome assembly protein 1-like 1	NAP1L1	1.64	0.069
NM_000433	Neutrophil cytosolic factor 2	NCF2	2.08	0.004

NM_003743	Nuclear receptor coactivator 1	NCOA1	1.43	0.086
NM_006169	Nicotinamide N-methyltransferase	NNMT	2.11	0.003
NM_000603	Nitric oxide synthase 3 (endothelial cell)	NOS3	1.64	0.077
NM_013392	Nuclear receptor binding protein 1	NRBP1	1.67	0.064
NM_003489	Nuclear receptor interacting protein 1	NRIP1	1.95	0.021
BC013770	Netrin G2	NTNG2	1.63	0.051
NM_000274	Ornithine aminotransferase	OAT	2.44	0.001
NM_019897	Olfactory receptor, family 2, subfamily S, member 2	OR2S2	1.62	0.059
NM_012387	Peptidyl arginine deiminase, type IV	PADI4	2.14	0.004
NM_005451	PDZ and LIM domain 7 (enigma)	PDLIM7	1.78	0.030
NM_020651	Pellino homolog 1 (Drosophila)	PELI1	1.52	0.022
NM_000318	Peroxisomal biogenesis factor 2	PEX2	2.13	0.010
NM_004566	6-phosphofructo-2-kinase/fructose-2,6-biphosphatase 3	PFKFB3	2.00	0.021
NM_000291	Phosphoglycerate kinase 1	PGK1	4.16	0.000
NM_005091	Peptidoglycan recognition protein 1	PGLYRP1	2.42	0.000
NM_024829	Phospholipase B domain containing 1	PLBD1	3.60	0.000
NM_012268	Phospholipase D family, member 3	PLD3	1.61	0.087
AF131859	Pallidin homolog (mouse)	PLDN	1.80	0.030
NM_021105	Phospholipid scramblase 1	PLSCR1	2.55	0.000
AF035307	Plexin C1	PLXNC1	1.96	0.021
NM_002709	Protein phosphatase 1, catalytic subunit, beta isozyme	PPP1CB	1.94	0.021
NM_021132	Protein phosphatase 3, catalytic subunit, beta isozyme	PPP3CB	1.68	0.066
NM_002720	Protein phosphatase 4, catalytic subunit	PPP4C	1.80	0.087
NM_000310	Palmitoyl-protein thioesterase 1	PPT1	1.76	0.049
NM_006445	PRP8 pre-mRNA processing factor 8 homolog (S. cerevisiae)	PRPF8	1.73	0.077
BI917904	Proline rich 5 (renal)	PRR5	1.65	0.067
NM_002777	Proteinase 3	PRTN3	1.76	0.002
NM_000952	Platelet-activating factor receptor	PTAFR	1.67	0.087
NM_002835	Protein tyrosine phosphatase, non-receptor type 12	PTPN12	1.88	0.081
NM_015967	Protein tyrosine phosphatase, non-receptor type 22 (lymphoid)	PTPN22	2.33	0.003
NM_002838	Protein tyrosine phosphatase, receptor type, C	PTPRC	2.30	0.001
NM_012413	Glutaminyl-peptide cyclotransferase	QPCT	2.66	0.000
NM_032846	RAB2B, member RAS oncogene family	RAB2B	1.80	0.080
NM_014857	RAB GTPase activating protein 1-like	RABGAP1L	1.75	0.099
NM_005053	RAD23 homolog A (S. cerevisiae)	RAD23A	1.75	0.032

AL049219	RNA binding motif protein 8A	RBM8A	1.79	0.021
BE737594	Recombination signal binding protein for immunoglobulin kappa J region	RBPJ	1.99	0.038
AJ006835	Regulator of chromosome condensation 1	RCC1	1.84	0.018
NM_018307	Ras homolog gene family, member T1	RHOT1	1.81	0.087
NM_002950	Ribophorin I	RPN1	1.86	0.042
AA381384	Ribosomal protein S29	RPS29	1.58	0.031
NM_005621	S100 calcium binding protein A12	S100A12	2.42	0.000
NM_002964	S100 calcium binding protein A8	S100A8	2.07	0.001
AA318707	S100 calcium binding protein A9	S100A9	2.14	0.001
NM_005980	S100 calcium binding protein P	S100P	2.55	0.000
NM_006745	Sterol-C4-methyl oxidase-like	SC4MOL	1.87	0.016
NM_016211	SEC31 homolog A (S. cerevisiae)	SEC31A	2.12	0.013
NM_003944	Selenium binding protein 1	SELENBP1	2.09	0.004
AL136807	Stress-associated endoplasmic reticulum protein 1	SERP1	4.17	0.000
NM_030666	Serpin peptidase inhibitor, clade B (ovalbumin), member 1	SERPINB1	2.27	0.002
AJ238403	SET domain containing 2	SETD2	1.97	0.021
AF258553	SH3KBP1 binding protein 1	SHKBP1	1.92	0.030
NM_030807	Solute carrier family 2 (facilitated glucose transporter), member 11	SLC2A11	2.15	0.000
NM_003098	Syntrophin, alpha 1 (dystrophin-associated protein A1, 59kDa, acidic component)	SNTA1	1.63	0.046
NM_004598	Sparc/osteonectin, cwcv and kazal-like domains proteoglycan (testican) 1	SPOCK1	1.72	0.068
NM_006374	Serine/threonine kinase 25	STK25	1.81	0.057
NM_005642	TAF7 RNA polymerase II, TATA box binding protein (TBP)-associated factor, 55kDa	TAF7	1.92	0.069
NM_003186	Transgelin	TAGLN	1.61	0.087
NM_006070	TRK-fused gene	TFG	1.97	0.022
NM_003217	Transmembrane BAX inhibitor motif containing 6	TMBIM6	2.02	0.021
L40391	Transmembrane emp24-like trafficking protein 10 (yeast)	TMED10	1.68	0.076
NM_007115	Tumor necrosis factor, alpha-induced protein 6	TNFAIP6	1.58	0.057
NM_003841	Tumor necrosis factor receptor superfamily, member 10c, decoy without an intracellular domain	TNFRSF10 C	1.66	0.086
NM_001068	Topoisomerase (DNA) II beta 180kDa	TOP2B	1.53	0.069
NM_001656	Tripartite motif-containing 23	TRIM23	1.94	0.026
J05428	UDP glucuronosyltransferase 2 family, polypeptide B7	UGT2B7	1.76	0.053
NM_018206	Vacuolar protein sorting 35 homolog (S. cerevisiae)	VPS35	2.05	0.016
X56196	X (inactive)-specific transcript (non-protein coding)	XIST	1.47	0.000
NM_021083	X-linked Kx blood group (McLeod syndrome)	XK	1.94	0.022

NM_004559	Y box binding protein 1	YBX1	1.93	0.064
NM_006336	Zer-1 homolog (C. elegans)	ZER1	2.20	0.004

Supplemental Table 1B (down regulated genes, FDR <0.1)

GenBank Acc.	Gene Name	Gene Symbol	Fold change	FDR
AF070569	Chromosome 17 open reading frame 91	C17orf91	-1.46	0.083
NM_017860	Chromosome 1 open reading frame 56	C1orf56	-1.90	0.083
NM_007293	Complement component 4A (Rodgers blood group)	C4A	-1.40	0.074
AF007146	Coiled-coil domain containing 57	CCDC57	-1.61	0.090
NM_002984	Chemokine (C-C motif) ligand 4	CCL4	-2.01	0.015
NM_000732	CD3d molecule, delta (CD3-TCR complex)	CD3D	-1.75	0.099
NM_005192	Cyclin-dependent kinase inhibitor 3	CDKN3	-1.77	0.089
NM_005666	Complement factor H-related 2	CFHR2	-1.49	0.099
NM_004267	Carbohydrate (N-acetylglucosamine-6-O) sulfotransferase 2	CHST2	-1.79	0.084
NM_001828	Charcot-Leyden crystal protein	CLC	-1.60	0.009
NM_016509	C-type lectin domain family 1, member B	CLEC1B	-1.70	0.036
NM_004718	Cytochrome c oxidase subunit VIIa polypeptide 2 like	COX7A2L	-2.06	0.021
NM_004380	CREB binding protein	CREBBP	-1.95	0.023
U20350	Chemokine (C-X3-C motif) receptor 1	CX3CR1	-2.22	0.005
NM_004660	DEAD (Asp-Glu-Ala-Asp) box polypeptide 3, Y-linked	DDX3Y	-1.59	0.005
NM_004746	Discs, large (Drosophila) homolog-associated protein 1	DLGAP1	-1.54	0.083
NM_004418	Dual specificity phosphatase 2	DUSP2	-1.92	0.009
NM_004681	Eukaryotic translation initiation factor 1A, Y-linked	EIF1AY	-1.02	0.000
AJ301564	Family with sequence similarity 167, member A	FAM167A	-1.94	0.009
NM_002001	Fc fragment of IgE, high affinity I, receptor for; alpha polypeptide	FCER1A	-2.20	0.003
AF250920	Forkhead box P1	FOXP1	-1.87	0.054
NM_014164	FXYD domain containing ion transport regulator 5	FXYD5	-1.12	0.029
AB033068	Fizzy/cell division cycle 20 related 1 (Drosophila)	FZR1	-1.84	0.091
M77140	Galanin prepropeptide	GAL	-1.66	0.001
NM_002051	GATA binding protein 3	GATA3	-1.84	0.043
AK055914	Guanine nucleotide binding protein (G protein), gamma 12	GNG12	-1.68	0.092
NM_012483	Granulysin	GNLY	-2.48	0.001
AL137763	Grainyhead-like 3 (Drosophila)	GRHL3	-1.88	0.009

NM_016545	Immediate early response 5	IER5	-1.65	0.089
NM_002180	Immunoglobulin mu binding protein 2	IGHMBP2	-2.23	0.005
NM_014425	Inversin	INVS	-1.21	0.022
NM_000216	Kallmann syndrome 1 sequence	KAL1	-1.79	0.003
NM_004653	Lysine (K)-specific demethylase 5D	KDM5D	-1.13	0.014
NM_014686	KIAA0355	KIAA0355	-1.90	0.090
AL050370	Kelch-like 35 (Drosophila)	KLHL35	-1.52	0.090
NM_002258	Killer cell lectin-like receptor subfamily B, member 1	KLRB1	-1.75	0.069
NM_002260	Killer cell lectin-like receptor subfamily C, member 2	KLRC2	-1.64	0.030
NM_007334	Killer cell lectin-like receptor subfamily D, member 1	KLRD1	-1.83	0.053
N57256	Lix1 homolog (chicken)	LIX1	-2.43	0.001
AK024496	Hypothetical LOC100129637	LOC10012 9637	-2.04	0.037
NM_032041	Neurocalcin delta	NCALD	-1.87	0.091
U60873	Nuclear paraspeckle assembly transcript 1 (non-protein coding)	NEAT1	-1.67	0.015
NM_004845	Phosphate cytidylyltransferase 1, choline, beta	PCYT1B	-1.73	0.015
AB023148	PH domain and leucine rich repeat protein phosphatase 2	PHLPP2	-1.93	0.027
NM_002638	Peptidase inhibitor 3, skin-derived	PI3	-2.18	0.009
AL117427	Plexin A4	PLXNA4	-1.82	0.092
NM_006347	Peptidylprolyl isomerase H (cyclophilin H)	PPIH	-1.80	0.089
AK000296	Proline rich 11	PRR11	-1.71	0.084
NM_033544	RCC1 domain containing 1	RCCD1	-1.77	0.074
BC015510	Regulator of G-protein signaling 1	RGS1	-1.96	0.001
NM_021194	Solute carrier family 30 (zinc transporter), member 1	SLC30A1	-1.89	0.071
AI929792	Synuclein, alpha (non A4 component of amyloid precursor)	SNCA	-1.66	0.037
NM_012445	Spondin 2, extracellular matrix protein	SPON2	-1.81	0.043
AB007925	SLIT-ROBO Rho GTPase activating protein 2	SRGAP2	-1.89	0.010
AL050041	Serine/arginine-rich splicing factor 3	SRSF3	-1.92	0.021
AF450267	Serine/threonine kinase 11 interacting protein	STK11IP	-1.83	0.099
NM_016140	Tubulin polymerization-promoting protein family member 3	TPPP3	-1.59	0.085
NM_014779	TSC22 domain family, member 2	TSC22D2	-1.70	0.100
NM_017742	Zinc finger, CCHC domain containing 2	ZCCHC2	-2.14	0.010
NM_030751	Zinc finger E-box binding homeobox 1	ZEB1	-1.72	0.007
NM_024833	Zinc finger protein 671	ZNF671	-2.69	0.000
NM_018335	Zinc finger protein 839	ZNF839	-1.72	0.089

Supplemental Table 2: Characteristics of validation cohort used for replication

	EOI	Non-EOI (control)
**n**	15	28
**GA (weeks)**	26.3 (23.6-30.6)	30.4 (24.3-31.7)
**Birth weight (g)**	810 (530-1650)	1420 (550-1970)
**ANCS**	3 (20%)	4 (14%)
**Chorioamnionitis**	2 (13%)	4 (14%)
**PROM**	5 (33%)	10 (36%)
**C-section**	3 (20%)	5 (18%)
**Blood culture positive**	0	0
**CRIB**	5 (1-8)	4 (1-9)
**RDS**	15 (100%)	24 (86%)
**RDS ≥ grade III**	0 (0%)	9 (32%)
**IVH**	9 (60%9	4 (14%)
**BPD**	10 (67%)	6 (21%)
**Length of mechanical ventilation (days)**	8 (3-34)	4 (2-30)
**ROP**	11 (73%)	5 (18%)
**Length of hospital stay (days)**	83 (36-242)	52 (27-136)
**Death**	0	0

Data are given as median and range or per cent of total in group.

EOI: early onset infection; GA: gestational age; IUGR: intrauterine growth restriction; PROM premature rupture of membranes; ANCS: antenatal corticosteroids: complete course including two doses of betamethasone given >24 hours prior to birth, last dose <7 days before birth; * any ANCS before birth; CRIB: critical risk index for babies; RDS: respiratory distress syndrome; IVH: intraventricular haemorrhage; BPD: bronchopulmonary dysplasia; ROP: retinopathy of prematurity

Supplemental Table 3: Amplification efficiencies of 10 target genes used for RT-PCR

Gene	Amplification efficiency*
1) GNLY	0.9814
2) KLRD1	0.9144
3) KLRC2	0.9731
4) CD163	0.9029
5) TNFRSF10A	0.9612
6) HIF1A	1.0282
7) ANXA1	0.9444
8) MPO	0.9641
9) PGLYRP1	0.9813
10) CD177	0.9221

* Amplification efficiencies for the target genes were calculated as E = 10(-1/S) -1, were S is the slope of the standard curve.

Supplemental Table 4: Top functional annotation cluster (Rank Products analysis)

Enrichment Score: 5.47		
**Term**	**p-value**	**Genes**
GO:0006952~defense response	6.05E-10	KYNU, KLRC2, S100A8, PGLYRP1, BNIP3, HP, ITGB1, CCL4, IL10, AZU1, FCGR1C, CXCR4, FCGR1A, GATA3, LTF, PTPRC, CEBPB, LYN, IL8, C4A, NCF2, GNLY, ANXA1, CHST2, GAL, HPR, S100A12, CD163, TNFAIP6, HIF1A, LILRB3, BNIP3L, CX3CR1, MPO, LTA4H, PTAFR, CLEC1B
GO:0006954~inflammatory response	1.54E-04	CEBPB, C4A, LYN, IL8, S100A8, CHST2, ANXA1, GAL, CCL4, IL10, S100A12, CD163, AZU1, TNFAIP6, HIF1A, CXCR4, LTA4H, PTAFR
GO:0009611~response to wounding	4.22E-04	PLDN, CEBPB, C4A, LYN, IL8, S100A8, ANXA1, CHST2, GRHL3, GAL, CCL4, IL10, S100A12, CD163, AZU1, TNFAIP6, PLSCR1, HIF1A, ADM, CXCR4, CX3CR1, LTA4H, PTAFR

Enrichment Score: 1.93		
**Term**	**p-value**	**Genes**
GO:0006935~chemotaxis	0.008	AZU1, IL8, CXCR4, KAL1, CX3CR1, FPR1, CCL4, IL10, PTAFR
GO:0042330~taxis	0.008	AZU1, IL8, CXCR4, KAL1, CX3CR1, FPR1, CCL4, IL10, PTAFR
GO:0006928~cell motion	0.027	S100P, IL8, FPR1, ANXA1, SPOCK1, ITGB1, CCL4, IL10, AZU1, HIF1A, CXCR4, PEX2, KAL1, NOS3, TOP2B, SPON2, CEACAM1

Enrichment Score: 1.82		
**Term**	**p-value**	**Genes**
GO:0042177~negative regulation of protein catabolic process	0.005	HSP90AB1, IRAK3, FLNA, IL10
GO:0009894~regulation of catabolic process	0.011	SPDYA, HSP90AB1, IRAK3, HIF1A, PPP1CB, FLNA, IL10, ADAM9
GO:0042176~regulation of protein catabolic process	0.017	HSP90AB1, IRAK3, FLNA, IL10, ADAM9
GO:0031329~regulation of cellular catabolic process	0.023	SPDYA, HSP90AB1, HIF1A, PPP1CB, IL10, ADAM9
GO:0009895~negative regulation of catabolic process	0.033	HSP90AB1, IRAK3, FLNA, IL10

Supplemental Table 5: Functional annotation cluster (Rank Products analysis with SVA correction)

Enrichment Score: 2.38		
**Term**	**p-value**	**Genes**
GO:0006952~defense response	5.71E-04	PTPRC, KLRC2, CEBPB, IL8, GNLY, PGLYRP1, HP, HPR, AZU1, CXCR4, GATA3, CX3CR1, BNIP3L, LTF, AKIRIN2
GO:0009617~response to bacterium	0.008	AZU1, ADM, GNLY, PGLYRP1, LTF, AKIRIN2
GO:0042742~defense response to bacterium	0.016	AZU1, GNLY, PGLYRP1, LTF
Enrichment Score: 2.04		
**Term**	**p-value**	**Genes**
GO:0006952~defense response	5.71E-04	PTPRC, KLRC2, CEBPB, IL8, GNLY, PGLYRP1, HP, HPR, AZU1, CXCR4, GATA3, CX3CR1, BNIP3L, LTF, AKIRIN2
GO:0006935~chemotaxis	0.036	AZU1, IL8, CXCR4, KAL1, CX3CR1
GO:0042330~taxis	0.036	AZU1, IL8, CXCR4, KAL1, CX3CR1
Enrichment Score: 1.21		
**Term**	**p-value**	**Genes**
GO:0007276~gamete generation	0.034	CXCR4, TMBIM6, DLD, AQP7, PCYT1B, KDM5D, NRIP1
GO:0019953~sexual reproduction	0.051	CXCR4, TMBIM6, DLD, AQP7, PCYT1B, KDM5D, NRIP1
GO:0032504~multicellular organism reproduction	0.092	CXCR4, TMBIM6, DLD, AQP7, PCYT1B, KDM5D, NRIP1
GO:0048609~reproductive process in a multicellular organism	0.092	CXCR4, TMBIM6, DLD, AQP7, PCYT1B, KDM5D, NRIP1

Supplemental Table 6: White blood count of EOI and non-EOI after imputation

White blood cells [x3/µl]	EOI			Non-EOI			p-value
	N	Mean	SE	N	Mean	SE	
LEU	16	6.34	0.18	8	5.90	0.30	0.951
segNEU	16	2.73	0.23	8	1.47	0.11	0.312
bandNEU	16	1.75	0.18	8	0.46	0.05	0.188
juvNEU	16	0.31	0.02	8	0.37	0.02	0.297
LYM	16	3.38	0.10	8	4.46	0.18	0.071
MON	16	0.79	0.07	8	0.55	0.04	0.976

LEU (leucocytes); segNEU (segmented neutrophils); bandNEU (band neutrophils); juvNEU (juvenile neutrophils); LYM (lymphocytes); MON (monocytes); SE (standard error); p-value from Wilcoxon's test
